# Modulating the gut-immune axis to alleviate rheumatoid arthritis: mechanistic insights and therapeutic potential of traditional Chinese medicine

**DOI:** 10.3389/fimmu.2025.1637942

**Published:** 2025-09-11

**Authors:** Jiaguo Zhan, Yu Fu, Zhanbiao Liu, Shaozhuo Zhang, Chongming Wu

**Affiliations:** ^1^ School of Chinese Materia Medica, Tianjin University of Traditional Chinese Medicine, Tianjin, China; ^2^ Laboratory Animal Center, Tianjin University of Traditional Chinese Medicine, Tianjin, China; ^3^ Tianjin Key Laboratory of Therapeutic Substance of Traditional Chinese Medicine, Tianjin University of Traditional Chinese Medicine, Tianjin, China

**Keywords:** rheumatoid arthritis (RA), traditional Chinese medicine (TCM), gut microbiota, immunomodulation, gut-immune axis

## Abstract

Rheumatoid arthritis (RA), a chronic autoimmune disorder marked by systemic inflammation and joint destruction, remains challenging to treat due to the limitations of conventional therapies, including side effects and diminishing efficacy. Emerging research underscores the gut-immune axis—a dynamic interplay between gut microbiota, immune responses, and inflammation—as a pivotal contributor to RA pathogenesis. Traditional Chinese Medicine (TCM), recognized for its established safety and accessibility, has been shown to synergistically alleviate symptoms of RA when used alongside conventional treatments, while significantly reducing drug-related toxicity. Pre-clinical models and clinical trials have demonstrated that TCM formulations, bioactive phytochemicals, and their metabolites can modulate the gut-immune axis by restoring gut microbiota balance and regulating immune-inflammatory pathways. This review summarizes the multi-target effects of TCM, including microbiota modulation and immune system regulation, and proposes a microbiota-centered therapeutic strategy for RA. Although the role of Traditional Chinese Medicine in regulating gut microbiota and immune modulation supports its clinical translatability, rigorous mechanistic studies remain essential to facilitate its integration into mainstream rheumatology treatment strategies. This involves research on its pharmacokinetic-pharmacodynamic characteristics, validation of microbiome-dependent mechanisms, and investigation into mechanisms involving microbial metabolites. By integrating millennia of empirical knowledge with cutting-edge systems biology, TCM presents a microbiota-centered holistic strategy for RA management.

## 1 Introduction

Rheumatoid arthritis (RA) is a chronic, systemic autoimmune disease characterized by synovial inflammation and hyperplasia, leading to cartilage and bone destruction, as well as systemic manifestations such as pulmonary, cardiovascular, skin, psychological, and skeletal disorders. RA arises from disrupted immune tolerance and sustained immune activation, driving inflammation and tissue remodeling (1). It affects approximately 0.46% of the global population, with a higher prevalence in industrialized countries (2). RA develops as a result of both genetic predispositions and environmental factors, such as specific gene variants like human leukocyte antigen-DR beta chain 1 (HLA-DRB1) and lifestyle triggers including smoking, pollutant exposure, and viral infections. Risk factors modulate gene expression via epigenetic mechanisms, contributing to disease onset and progression. These factors can influence post-transcriptional modifications (PTMs) of specific genes or affect susceptibility genes via epigenetic mechanisms. The burden of RA is substantial due to its recurrent nature and high disability rate (1, 3).

The human gut microbiota (GM) represents a complex and dynamic ecosystem of microorganisms residing within the gastrointestinal tract (4). This microbial community includes diverse subgroups of bacteria, viruses, fungi, and archaea, all coexisting within the gastrointestinal environment. The gastrointestinal tract hosts a substantial proportion of the body’s immune cells and continuously interacts with the GM, thereby shaping their functions and properties (5). The gut microbiome, which includes microbiota, microbial structural components such as nucleic acids, metabolites, and environmental factors, plays a fundamental role in the priming and development of the immune system (6). The GM serves as an innate immune modulator, drug and diet metabolizer, and producer of biologically active metabolites. It is vital for modulating immune cell activities and inflammatory cytokines, thus helping to maintain balanced immune responses (5).

Increasing evidence and reports have demonstrated that there is an intricate and dynamic interaction between the GM and the immune system, forming what is known as the gut-immune axis (7–9). Numerous studies highlighted a critical role of the gut-immune axis in the pathogenesis of RA (5, 10–12). Dysbiosis of specific bacterial lineages and metabolic alterations in gut microbiota resulted in modifications to the host immune profile, which contribute to the development of RA (13). Extensive investigations have demonstrated that GM composition on fecal samples differs between RA patients and healthy controls (HCs), implying gut dysbiosis may contribute to RA pathogenesis (14–17). Recent research highlights that dysbiosis and compositional variations of GM in RA patients are key factors contributing to abnormal systemic immunity (18–20). It has been suggested that the mechanism through which gut dysbiosis leads to RA might be associated with the regulation of immune function by metabolites generated by GM (21–24). Intestinal barrier dysfunction precedes RA, which further supports the “gut-immune axis” in RA pathogenesis (25–27).

Despite efforts to develop anti-RA drugs, there is no safer and more sustainable therapeutic agent for RA in humans. Conventional treatments for RA include glucocorticoids (GCs), non-steroidal anti-inflammatory drugs (NSAIDs), and disease-modifying anti-rheumatic drugs (DMARDs). etc. NSAIDs, DMARDs and GCs could effectively relieve the pain of RA patients and inhibit the inflammatory reaction *in vivo*, but they fail to restore the native function of joints. Moreover, current therapeutic options are limited by deleterious side effects, high costs, inadequate control of disease progression in many patients, and diminishing therapeutic efficacy over time.

Traditional Chinese medicines (TCMs) have been used to treat various diseases since ancient times and shown to be safe and accessible to the general population in treating RA (28–30). Accumulating evidence have revealed that TCMs, their extracts, and bioactive compounds have anti-inflammatory, cartilage-protective, and immunoregulation properties and exhibit promising anti-RA activities (31–33). Clinical studies show TCMs are more effective with fewer side effects compared to conventional treatments. Combining TCM with synthetic DMARDs can reduce adverse effects of conventional therapies (34–39). TCMs offer advantages in modulating the gut-immune axis through multi-target regulation and lower toxicity (39–44). Notably, DMARDs such as methotrexate and leflunomide often cause gastrointestinal toxicity, whereas TCM can mitigate toxicity (45, 46).

However, due to their complex compositions and multiple targets, TCMs necessitate further investigation to elucidate the active ingredients and mechanisms of action in treating RA. Natural products derived from TCMs, characterized by their remarkable chemical diversity and bioactivity, hold significant potential as a foundation for developing novel pharmacological agents for RA treatment (47). Therefore, upon validation of their pharmacological potential, these TCM-derived natural products may provide promising leads for the development of modern anti-RA drugs.

Investigating the mechanisms by which TCMs regulate the gut-immune axis in RA treatment holds significant importance, as this identifies potential target for developing RA therapeutics. Consequently, this paper provides a comprehensive review of TCMs with anti-RA activities that specifically target the gut-immune axis, thereby paving the way for future research and development endeavors.

## 2 Overview of immune response in RA

RA pathogenesis is initiated by PTMs, such as citrullination, carbamylation, and glycosylation, which generate neoepitopes recognized as autoantigens. Citrullination, mediated by peptidyl arginine deiminases (PADs), converts arginine to citrulline, triggering anti-citrullinated protein antibody (ACPA) production. Genetic susceptibility enables T-cell recognition of modified peptides and disrupts T-cell signaling, promoting autoimmunity (1).

**Figure 1:**
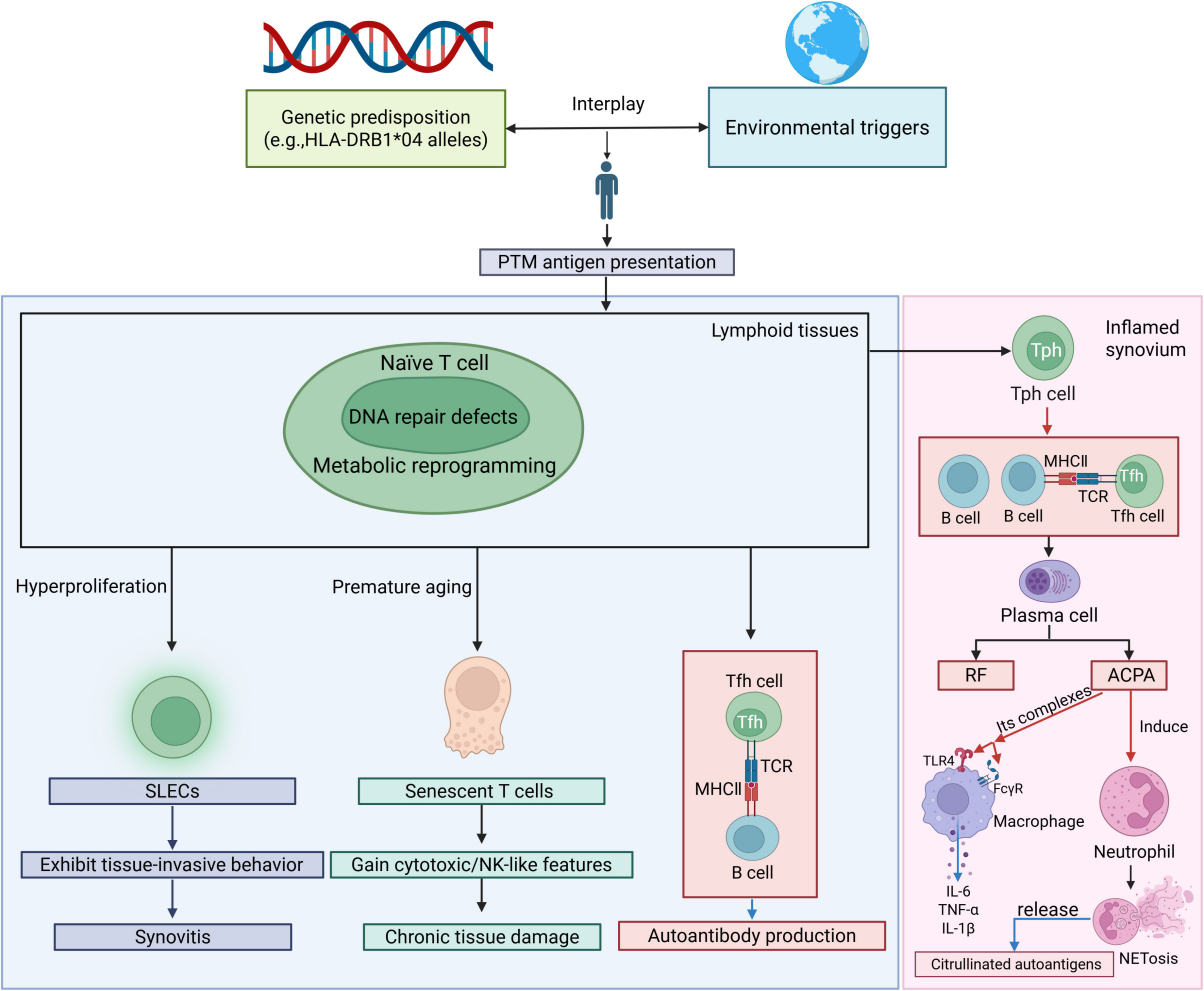
The crosstalk between T cells, neutrophils, and macrophages creates a vicious cycle in RA. SLECs, short-lived effector T cells; PTM, posttranslational modification; TCR, T cell receptor; MHC, major histocompatibility complex; RF, rheumatoid factor; ACPA, anti-citrullinated protein antibody; TLR, Toll-like receptor; FcγR, Fc gamma receptor. Red arrows indicate activation, facilitation or stimulation, whereas blue arrows represent the secretion of cytokines or release of autoantigens/autoantibodies.

**Figure 2:**
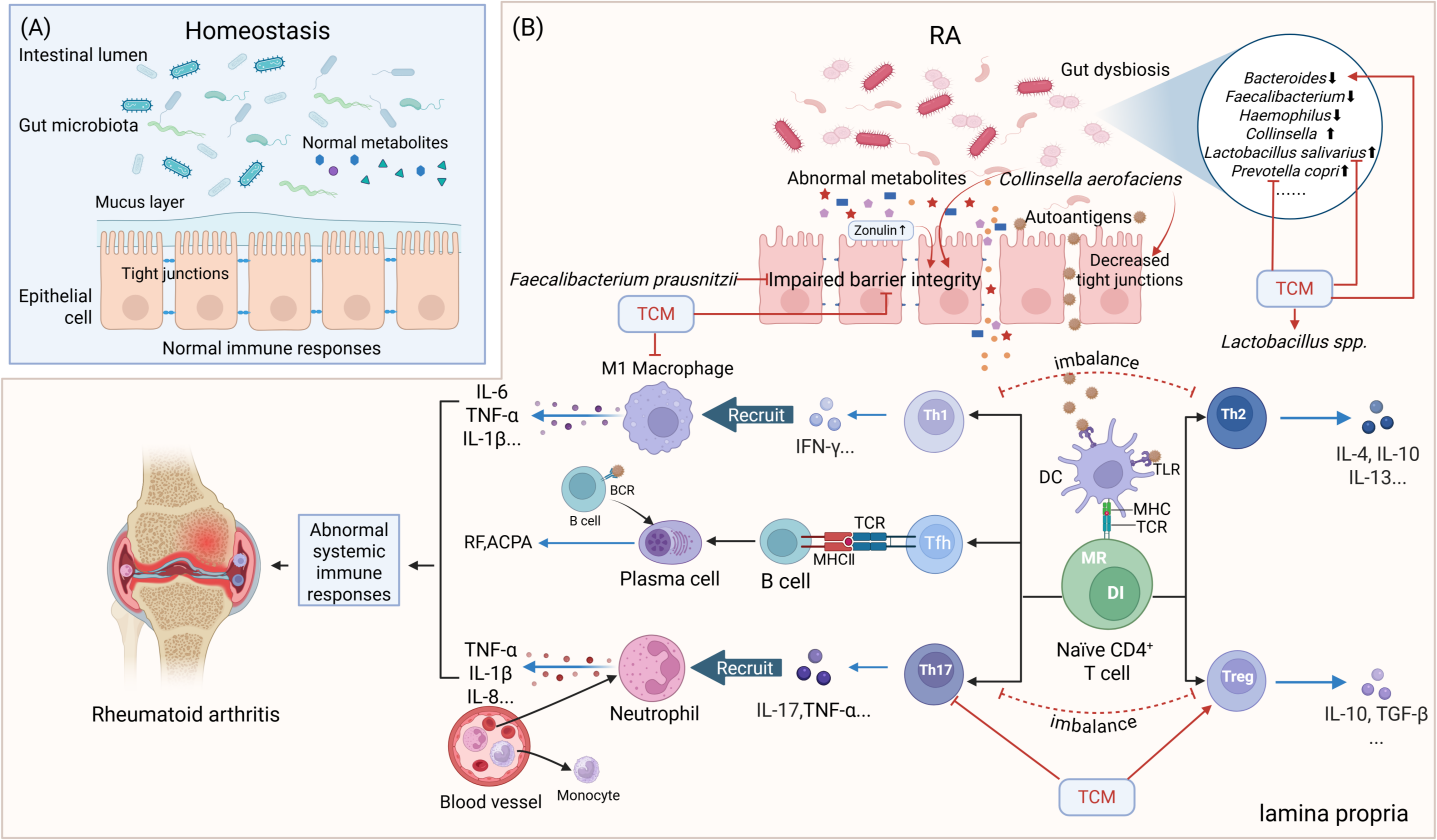
Simplified scheme of the gut-immune axis in the pathogenesis of RA and its TCM modulation. **(A)** The normal gut microbiota and their metabolites maintain the integrity of the intestinal epithelial cell layer and the homeostasis of gut immunity. **(B)** Impact of gut dysbiosis on gut barrier integrity and immune responses in RA and TCM interventions. Elevated Zonulin secretion leads to impaired gut barrier integrity. APCs recognize autoantigens and present them to T and B lymphocytes within lymphoid tissues, triggering an autoimmune response, ultimately leading to RA. DC=dendritic cell, BCR, B-cell receptor; DI, DNA instability; MR, Metabolic reprogramming. Red arrows indicate activation or facilitation, whereas red blocked lines indicate inhibition. Blue arrows represent the secretion of cytokines/autoantibodies.

Antigen-presenting cells (APCs) present these autoantigens to autoreactive T cells. Metabolic reprogramming and DNA repair defects drive abnormal T-cell differentiation into short-lived effector T cells (SLECs), contributing to premature senescence and skewed differentiation into proinflammatory subsets at the expense of regulatory T (Treg) cells and T helper-2 (Th2) cells. Senescent T cells acquire cytotoxic/NK-like properties, resisting apoptosis and sustaining inflammation (1, 48, 49) (**Figure 1**). Proinflammatory subsets include Th1, Th17, follicular helper T cells (Tfh) and peripheral helper T cells (Tph). Th1 cells produce interferon (IFN)-γ, tumor necrosis factor (TNF)-α, and interleukin (IL)-2, and amplify macrophage activation (50), whereas Th2 cells generally secrete L-4, IL-10, and IL-13, cytokines, and reduce macrophage activation (51). Th17 cells release proinflammatory cytokines such as IL-17, IL-21, and TNF-α, which affect chondrocytes, fibroblasts, osteoclasts, and neutrophils (52). Chondrocytes undergo apoptosis and pyroptosis and can be induced to release pro-inflammatory proteins, such as TNF-α, IL-6, collagenolytic enzymes, and matrix metalloproteinases (MMPs) (53). Treg cells, which secrete anti-inflammatory cytokines such as IL-10 and transforming growth factor- (TGF-) β1, are essential in controlling RA (54). Abnormal Th1/Th2 and Th17/Treg ratio have been detected in RA patients (55, 56) (**Figure 2**). Tfh and Tph cells expand in synovium, supporting B-cell maturation and autoantibody diversification (e.g., IgG ACPA) (1, 57, 58) (**Figure 1**).

Autoreactive B cells, stimulated by Tph/Tfh-derived chemokine (C-X-C) motif ligand 13 (CXCL13) and IL-21, differentiate into plasma cells secreting ACPA, rheumatoid factor (RF), and anti-PAD4 antibodies. B cells also secrete proinflammatory cytokines (IL-6, TNF-α), sustaining synovitis and ectopic lymphoid structures formation in joints (49, 57).

**Figure 3:**
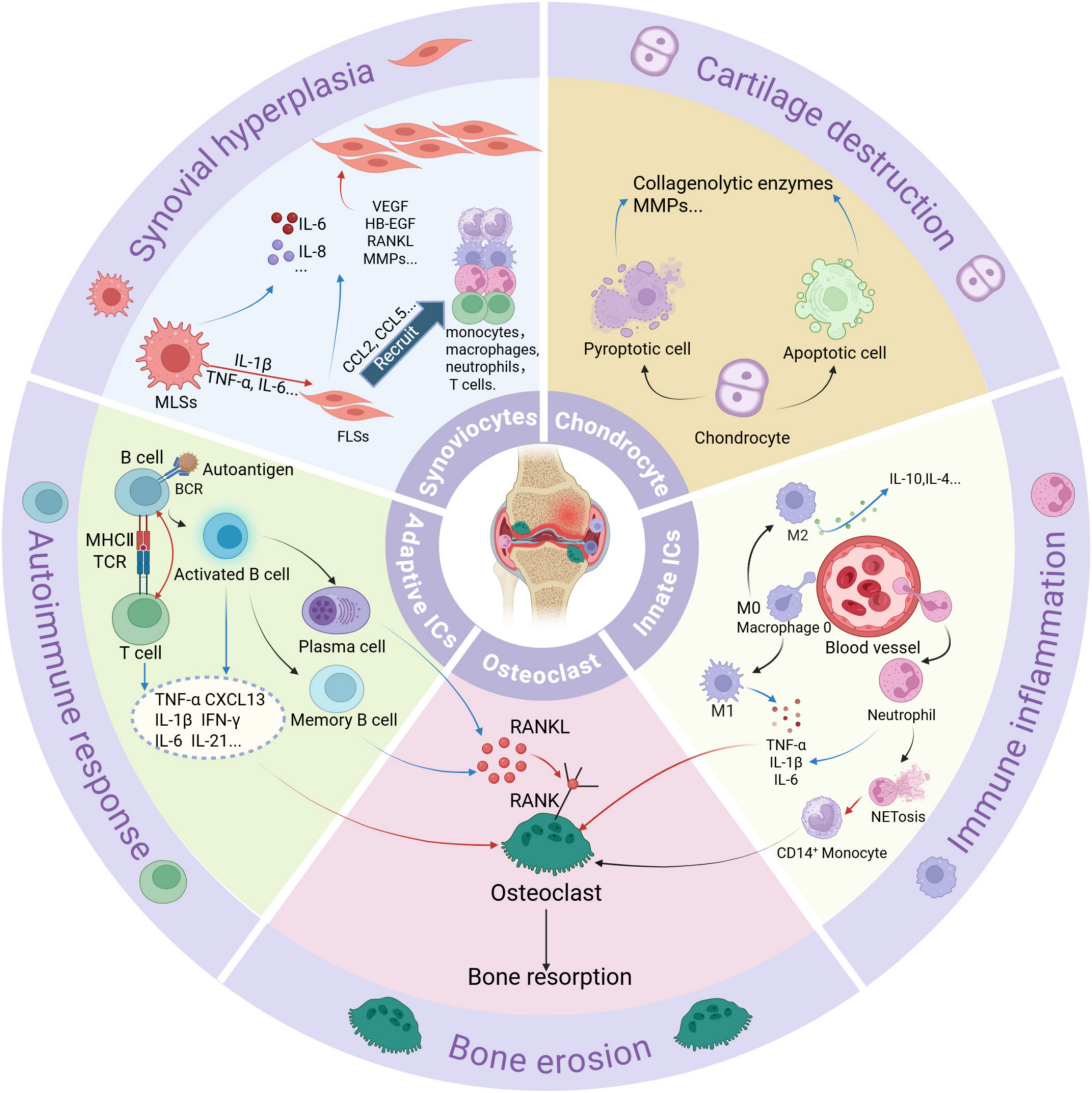
A summary of different cell types and their functions in RA. ICs, immune cells; RANKL, receptor activator of nuclear factor κB ligand. Red arrows indicate stimulation or activation. Blue arrows represent the secretion of pro-inflammatory proteins.

Macrophages, synoviocytes and neutrophils play important roles in RA innate immune activation and joint destruction. M1-polarized macrophages dominate RA synovium, releasing TNF-α, IL-1β, and MMPs that drive cartilage degradation. M2 macrophages, which secrete anti-inflammatory cytokines such as IL-4, IL-10, and TGF-β, are critical for tissue repair, become depleted, thereby impairing the resolution of inflammation (1, 59). Macrophage-like synoviocytes (MLSs) produce such cytokines as IL-1β, IL-6, and TNF-α to stimulate Fibroblast-like synoviocytes (FLSs) to secrete MMPs and receptor activator of nuclear factor κB ligand (RANKL) (60, 61). FLSs acquire an invasive phenotype, secret cytokines (e.g., IL-6, IL-17, and IL-33) and chemokines (e.g., C-C motif ligand 2/CCL2), and recruit immune cells such as monocytes/macrophages, neutrophils, and T cells. Moreover, these cells release growth factors and pro-angiogenic factors, such as vascular endothelial growth factor (VEGF) and heparin-binding epidermal growth factor-like growth factor (HB-EGF), thereby promoting FLS invasiveness, macrophage activation, angiogenesis, and sustaining synovial hyperplasia (61). Neutrophils produce pro-inflammatory proteins and neutrophil extracellular traps (NETs), which release citrullinated antigens and induce CD14+ monocytes to differentiate into osteoclasts through a RANKL-independent pathway (**Figure 1**). ACPAs further directly activate neutrophils and induce NETosis (NETs) (62, 63). Citrullinated fibrinogen-ACPA complexes in the RA synovium synergistically activate macrophages through dual engagement of Toll-like receptor 4 (TLR-4) and Fc gamma receptors (FcγR). This co-stimulation triggers robust TNF-α production (64, 65) (**Figures 1**, **3**).

In summary, a self-reinforcing cycle of innate-adaptive crosstalk, cytokine storms, and tissue destruction underpins RA progression.

## 3 The gut-immune axis in RA

The gut-immune axis represents a burgeoning concept that elucidates the bidirectional interactions between the gut microbiome and the immune system. Accumulating evidence highlights the critical role of the gut-immune axis in the pathogenesis of RA (10, 39, 48, 66). This axis operates through four primary mechanisms: (1) gut dysbiosis-driven immune dysregulation, (2) microbial metabolite-mediated immunomodulation, (3) intestinal barrier dysfunction, and (4) molecular mimicry of autoantigens. Below, we summarize current evidence linking these mechanisms to RA progression (**Figure 2**).

GM and their metabolites contribute to RA development through immunomodulatory effects. Gut dysbiosis, characterized by alterations in microbial diversity and abundance, is linked to RA pathogenesis in both patients and animal models (22, 67–71). The GM generates a variety of metabolites, including trimethylamine N-oxide, tryptophan derivatives, short-chain fatty acids (SCFAs), indole-3-acetate, bile acids, peptidoglycan, amines, polyamines, vitamins, and other small molecules (72). A growing body of evidence indicates that these microbial metabolites possess immunomodulatory properties and affect the development of RA (14, 73–75).

### 3.1 Gut dysbiosis in RA: microbial shifts and pathogenic drivers

Gut dysbiosis contributes to the occurrence of RA in both patients and animal models, with increased prevalence of *Prevotella* spp. in pre-clinical and diagnosed RA cases. While multiple *Prevotella* species other than *P. copri* are associated with RA etiology, *P. copri* itself is most abundant in new-onset RA and correlates with reduced *Bacteroides fragilis* levels (14, 71).

Animal models do not fully replicate human RA, but they provide valuable mechanistic insights despite differences in GM (12, 76). SKG mice develop arthritis when colonized with *Prevotella*, while germ-free or antibiotic-treated mice remain disease-free. Collagen-induced arthritis (CIA) mice show altered GM composition with reduced Bacteroidetes and increased Firmicutes and Proteobacteria during early arthritis onset (77). Germ-free (GF) L-1 receptor antagonist (IL-1Ra) knockout mice do not develop arthritis unless colonized with *Lactobacillus bifidus*, which induces rapid disease onset similar to conventional mice (78).

Additionally, GM affects the development of RA. Early RA patients show higher levels of *Lactobacillus* and *Blautia gnavus*, while *Acetanaerobacterium elongatum, Cristiansella massiliensis, and Gracilibacter thermotolerans* were significantly enriched in the control group (79, 80). TNF transgenic (TNF-Tg) mice overexpress human TNF-α, leading to spontaneous arthritis similar to human RA. Key mechanisms include TNF-α-driven inflammation via the Nuclear Factor kappa B (NF-κB) and Mitogen-activated protein kinase (MAPK) pathways, synovial hyperplasia, and bone erosion. These mice show increased *Prevotella*, *Aerococcus*, and *Staphylococcus* but reduced *Parasutterella* and *Clostridium_XIVa*. Dysbiosis promotes systemic inflammation via altered metabolites and increased gut permeability (81).

During the active phase of RA patients, *Haemophilus* and *Bacteroides* were reduced, while *Lactobacillus salivarius*, *Streptococcus*, *Akkermansia*, *Klebsiella*, and *Escherichia coli* were increased (21, 82, 83). Probiotic genera such as *Faecalibacterium* are decreased, while pathogenic bacteria including *Porphyromonas gingivalis*, *Collinsella*, and *Aggregatibacter actinomycetemcomitans* are more abundant in RA (83–85).

Taken together, these microbial shifts disrupt immune and metabolic homeostasis, contributing to the onset and exacerbation of autoimmunity. The findings highlight GM as a critical therapeutic target, emphasizing the need to restore microbial balance to attenuate RA progression.

### 3.2 Interactions between the GM and the immune system in RA

Substantial evidence indicates that gut dysbiosis in RA is a key factor contributing to systemic immune dysregulation. It is plausible that local tissue stress induces PTMs of peptides, which subsequently trigger antibody formation, serving as a common mechanism in RA (86). Certain GM such as *Collinsella* and *Porphyromonas gingivalis* encode functional microbial PADs which can leak into the human intestinal epithelium under conditions of increased intestinal permeability, leading to citrullination of peptides within the human gut. Citrullinated peptides from both human and bacterial proteins trigger loss of immune tolerance, especially in genetically predisposed individuals (85). For example, *Aggregatibacter actinomycetemcomitans* activates citrullinating enzymes in neutrophils, promoting autoantigen citrullination in RA joints. Specific citrullinated antigens such as vimentin, fibrinogen-alpha, and actin are targeted by ACPAs, suggesting the colon mucosa as a potential site for autoimmunity initiation (87). The Pc-p27 protein, a citrullinated peptide from *Prevotella copri*, induces Th1 immune responses in RA patients via binding to human leukocyte antigen (HLA)-DR (25). This association is further supported by the presence of IgA antibodies against Pc-p27 in both acute and chronic RA patients, which are linked to the production of Th17 cytokines and ACPA.

Autoantigens can be presented to CD4+ T cells by dendritic cells (DCs) and macrophages, driving inflammatory T cell differentiation and disrupting the Th17/Treg balance. Th17 cells promote B cell activation and antibody production, while Treg cells maintain immune tolerance and homeostasis by suppressing aberrant immune responses. *Lactobacillus* and *Bifidobacterium infantis* exert anti-inflammatory effects by inducing the expansion of Treg cells (88). The Th17/Treg ratio is significantly increased in advanced RA patients, highlighting the role of GM and metabolites in modulating this imbalance (10, 89) (**Figure 2**). *Lactobacillus bifidum* exacerbated arthritis by promoting Th17 and Th1 responses via TLR2/TLR4 signaling (78). *Lactobacillus plantarum strain TIFN101* enhances intestinal mucosal immunity by increasing IL-17-producing memory Th cells and upregulating major histocompatibility complex (MHC)-IIa expression (90). Moreover, Lactobacillus helveticus SBT2171 suppresses T/B cell proliferation and lymphoma cell cycle progression through JNK pathway inhibition *in vitro* (91). The phylum *Firmicutes* was negatively correlated with Th17 cell counts, while Verrucomicrobiota (e.g., *Akkermansia muciniphila*) were positively correlated with Treg numbers (13). Additionally, the accumulation of Treg cells in the colonic lamina propria can also be induced by *Clostridia* (92).

In contrast, the colonization of *Bacteroides fragilis* is associated with increased activity of regulatory T (Tregs), potentially mitigating the severity of autoimmune diseases (93, 94). The reduction in *Bacteroidetes* in CIA mice is thought to impair the differentiation of CD4+ T cells into Tregs, thereby contributing to an overall pro-inflammatory environment (95).

GF mice serve as a powerful and widely utilized model for investigating the impact of the microbiome on the immune system. *Segmented Filamentous Bacteria* (SFB) monocolonization in GF K/BxN mice induces autoantibody production, pathogenic Th17 cells, and arthritis (96). Additionally, SFB promotes Th17 cell accumulation in the gut via DC-presented antigens and IL-1β secretion induced by reactive oxygen species (ROS) (97, 98). SFB can induce autoimmune arthritis by promoting the differentiation and migration of gut Tfh to systemic lymphoid tissues, increasing autoantibody production (99). In contrast, depletion reduces Tfh cells and antibody levels, indicating that microbiota regulate arthritis via Tfh cells independently of Th17 cells (100).

Other studies employed the K/BxN model, in which mice co-expressed the T-cell receptor (TCR) transgene KRN and MHC class II molecule A (g7), leading to the development of autoantibodies against glucose-6-phosphate and subsequent severe inflammatory arthritis. GF conditions markedly reduce arthritis severity due to lower autoantibody levels and fewer Th17 cells (101). *Prevotella* and *Monoglobus* abundance correlates positively with Th1/Th2 cell counts and cytokine levels including IL-4, IL-2, IL-10, TNF-α, and IFN-γ (13).

Disrupted GM can also interact with other kinds of immune cells and their cytokines to modulate immune responses and inflammatory reactions, contributing to RA. Injection of *colonic E. coli* or *Enterococcus* into autoimmune-prone Dark Agouti rats caused a reduction in macrophages, an increase in activated neutrophils, and inflammatory polarization of peritoneal cells (102). Tanoue et al. found 11 bacterial strains, including *Bacteroides clarus 82C1*, *Bacteroides uniformis st. mat-281 81A2*, *Anaerostipes caccae 81B4*, *Bacteroides eggerthii 82B11*, *Bacteroides fragilis 82A12*, *Bacteroides cellulosilyticus 82B7*, *Bacteroides salyersiae 82A3*, *Clostridium* sp. *AUH-JLC39 82D29*, *Hungatella hathewayi 81G1*, *Clostridium* sp. *AT5 83F2*, and *Clostridium innocuum 81A1*, from healthy human donor faces that could induce IFN-γ-producing CD8+ T cells without intestinal inflammation (103).

**Figure 4:**
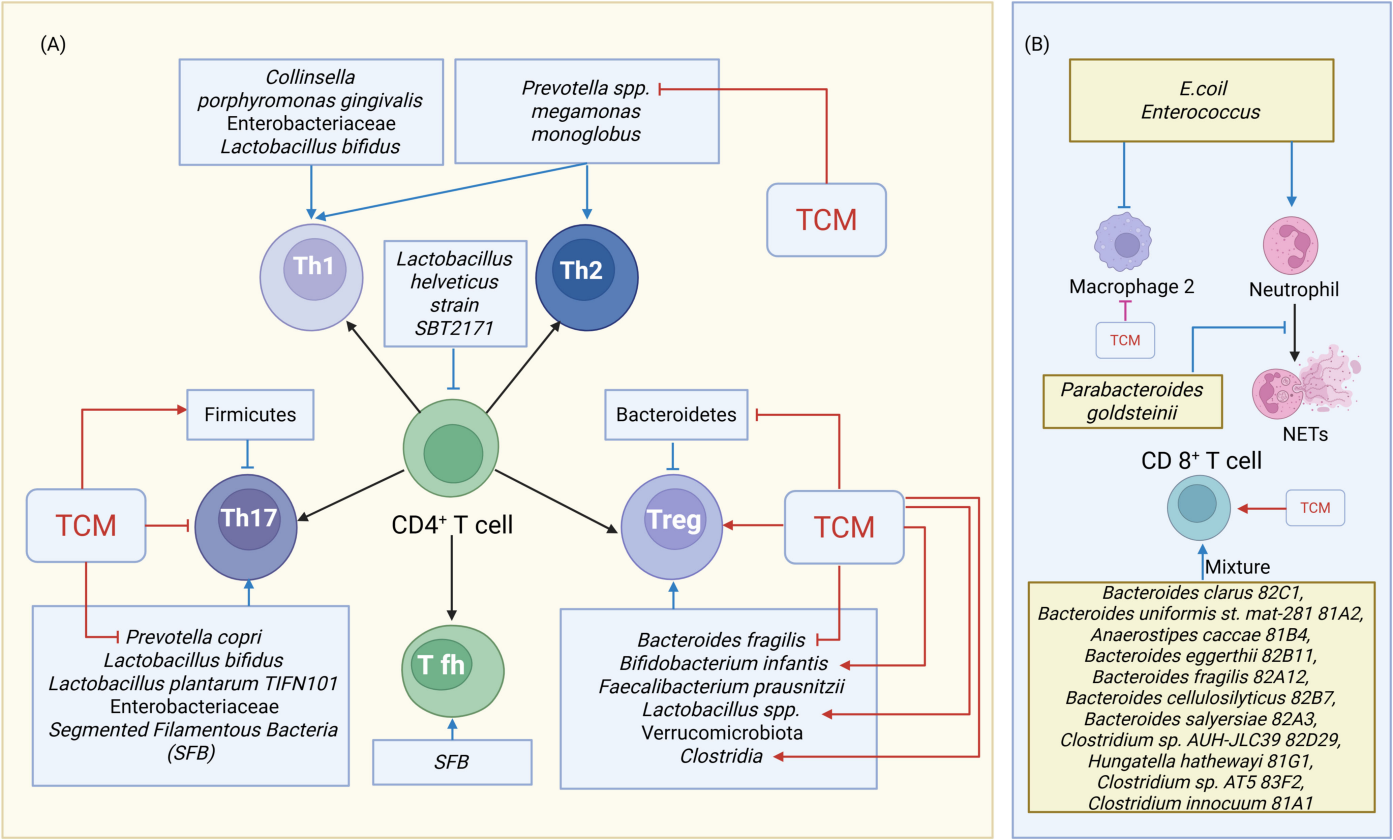
TCM’s Modulation of Disrupted Gut Microbiota and Immune Cell Crosstalk. **(A)** TCM’s Modulation of Gut Microbiota and T Cell Crosstalk. **(B)** TCM’s Modulation of Gut Microbiota and other immune Cell Crosstalk. Blue indicators represent the actions of gut microbiota on immune cells, while red indicators represent the effects of TCM on immune cells or gut microbiota. Arrows indicate activation or facilitation, whereas blocked lines indicate inhibition. NETs=neutrophil extracellular traps.

A novel intestinal immune regulatory pathway involves macrophage sensing of microbes via myeloid differentiation primary response 88 (MyD88) and Nucleotide-binding oligomerization domain 2 (Nod2), leading to IL-1β production and innate lymphoid cells (ILC) 3-derived IL-2, essential for intestinal Treg induction (104). Clinically, the abundance of *P. goldsteinii* correlates negatively with NETs indices and RA disease activity (105). CD8+ T cells displayed notable alterations in RA patients characterized by dysregulation of both *Prevotella* and *Bacteroides* microbiota (106) (**Figure 4**).

The overactivation of Th1 cells and Th17 cells, induced by a disrupted GM, results in the excessive production of pro-inflammatory cytokines such as TNF, IL-6, and IL-17. This triggers systemic inflammation and immune dysregulation, playing a critical role in autoimmune diseases like RA. Specifically, the disruption of GM within the *Enterobacteriaceae* family activates the NF-κB signaling pathway, promoting the release of pro-inflammatory cytokines and thereby contributing to inflammation (107).

In contrast, some strains, such as *Lactobacillus casei* and *Lactobacillus acidophilus* exhibit significant anti-inflammatory and antioxidant effects, protecting against CIA (108). *Faecalibacterium prausnitzii* induces the secretion of IL-10 by CD4+ T cells and exhibits substantial anti-inflammatory effects (109). A previous study indicated that *L. helveticus* SBT2171 could up-regulate the expression of A20, a negative regulator of NF-κB/MAPK signaling, via TLR2 signaling, thereby suppressing IL-6 and IL-1β production by APCs (110).

Collectively, the interplay between gut dysbiosis and RA pathogenesis is underscored by mechanisms linking microbial activity to systemic immune dysregulation. These findings highlight the therapeutic potential of targeting GM through probiotics, dietary interventions, or microbial transplants to restore immune balance. However, the complexity of microbial-immune interactions necessitates further research to delineate strain-specific effects and optimize translational strategies for RA management.

### 3.3 Interactions between the gut microbial metabolite and the immune system in RA

Dysfunctional GM can lead to alterations in fecal metabolites and compromise gut barrier integrity, permitting metabolites to enter the circulatory system, thereby inducing inflammatory processes and immune responses (23, 24). The primary SCFAs produced by GM in the human gut are acetate, propionate, and butyrate. Other SCFAs include pentanoate, hexanoate, and heptanoate (111). The concentrations of acetate, propionate, butyrate, and valerate were found to be reduced in RA patients (22, 80, 112). These SCFAs correlate positively with B cell frequency and can inhibit B cell differentiation and autoantibody production (113). Some immunomodulatory properties of SCFAs are attributed to their influence on both innate and adaptive immune system cells through the inhibition of histone deacetylases (HDACs) (114). Specifically, SCFAs enhance IL-10 production in T-helper 1 cells via the G protein-coupled receptor 43 (GPR43) pathway and inhibit HDAC activity during T helper 1 and Th17 differentiation (115). They also stimulate IL-22 production in CD4+ T cells through a GPR41-dependent pathway and reduce HDAC activity (115). SCFAs play crucial roles in regulating the balance between anti-inflammatory Tregs and pro-inflammatory Th17 cells by targeting key transcription factors. They promote Treg differentiation through multiple mechanisms. Butyrate inhibit HDACs, increasing histone acetylation at the Forkhead box P3(Foxp3) promoter. This enhances the transcription of Foxp3 which is the master transcription factor for Tregs (72). Additionally, SCFAs bind to GPR43 and GPR109A, inhibit HDAC, activate signal transducer and activator of transcription 3 (STAT3) signaling pathways, and boost Foxp3 expression (72, 116, 117). Moreover, SCFAs induce retinal dehydrogenase, facilitating the conversion of vitamin A into retinoic acid, which promotes Treg differentiation (72). In contrast, SCFAs suppress Th17 cell activity. For instance, butyrate inhibits retinoic acid-related orphan receptor gamma t (RORγt) via HDAC inhibition and IL-6/STAT3 blockade, reducing Th17 gene expression (117).

Butyrate-treated DCs enhance Treg differentiation and suppress Th1 cell differentiation by upregulating the expression of immunosuppressive enzymes, including indoleamine 2,3-dioxygenase 1 and aldehyde dehydrogenase 1 family member A2 via an SLC5A8-dependent mechanism. SCFAs have been shown to regulate neutrophils and macrophages, thereby modulating the intensity of inflammatory responses (118–120). Specifically, acetate and propionate activate the cell surface receptor GPR43, promoting neutrophil chemotaxis (121). SCFAs promote M2 macrophage polarization and reduce pro-inflammatory cytokine expression (122). They also maintain colonic Treg homeostasis, reduce B cell IgG, IgA, and IgE secretion, and suppress plasma cell differentiation (72). SCFAs correlate with increased Tregs and decreased IL-17A, IL-6, and TNF-α in CIA rats, and their administration alleviates arthritis severity by expanding Foxp3+ IL-10+ Tregs (123, 124). Furthermore, the production of SCFAs is proposed as one of the mechanisms through which GM influences Treg cell differentiation (125).

SCFAs block NF-κB via HDAC inhibition or peroxisome proliferator-activated receptor gamma (PPARγ) activation and exert anti-inflammatory effects. This leads to reduced expression of inflammatory mediators such as cytokines, chemokines, inducible nitric oxide synthase (iNOS), cyclooxygenase-2 (COX-2), and adhesion molecules (126, 127). Butyrate specifically decreases LPS-induced proinflammatory mediators like nitric oxide (NO), IL-6, and IL-12 in macrophages (128). These cytokines enter circulation and affect the joints.

Microbial tryptophan metabolites, such as indoles and their derivatives, engage with aryl hydrocarbon receptors (AhRs) to influence B cell development, differentiation, cytokine production, and regulation via AhR signaling pathways. Furthermore, bile acids and their metabolites modulate immune responses by regulating signaling pathways and maintaining the balance between Th17 and Treg cells (11). The bile acids derived from live *P. distasonis* (LPD), including lithocholic acid (LCA), deoxycholic acid (DCA), isolithocholic acid (isoLCA), and 3-oxolithocholic acid (3-oxoLCA), exhibited both similar and synergistic effects in mitigating RA. Notably, 3-oxoLCA and isoLCA not only directly inhibited the differentiation of Th17 cells but were also identified as TGR5 agonists that promoted the M2 polarization of macrophages. Furthermore, a specific synthetic inhibitor of bile salt hydrolase diminished the antiarthritic effects of LPD by reducing the production of these four bile acids (129).

LPS activates TLR4 and the NF-κB pathway, triggering inflammation and activating the complement alternative pathway, which contributes to arthritis (129, 130). *Bacteroides fragilis* secretes polysaccharide A (PSA), which stimulates Th1 responses, affects epithelial IL-17A production (15), corrects systemic T cell deficiencies, restores Th1/Th2 balance, and promotes lymphoid organogenesis (131). Colonization with *Bacteroides* in GF mice increases the population of Tregs via CD4+ T cell stimulation by PSA (93, 132).

In conclusion, GM metabolites (SCFAs, BAs, tryptophan derivatives) are critical regulators of immune cells, especially T cell subsets. Their dysregulation in RA disrupts the Th17/Treg equilibrium, driving inflammation and joint damage. Targeting these metabolites offers promising strategies to restore immune balance and mitigate RA progression.

### 3.4 Intestinal barrier dysfunction

The gut mucosal barrier, comprised of a monolayer of intestinal epithelial cells interconnected by tight junctions (TJ), separates the host from dietary and microbial antigens. Zonulin regulates TJ function by altering the expression of proteins like Zonula Occludens-1, occludin, claudin-1, claudin-2, and claudin-15, increasing intestinal permeability (20). In murine models, elevated zonulin levels lead to TJ disruption, promoting T-cell-mediated inflammation and migration of autoreactive Th1/Th17 cells from the gut to joints, contributing to RA development (133). Zonulin antagonists such as larazotide acetate reduce arthritis onset in mice (26). Flak et al. found increased gut permeability due to reduced numbers of TJ compared to HCs (27).

The gut integrity is compromised in RA patients, resulting in translocation of microbiota or their metabolites across the gut barrier into the lamina propria. The interaction between TLRs and pathogen-associated molecular patterns on these microbes can potentially activate the immune system, inducing pro-inflammatory cytokines like IL-6, TNF-α, or IL-1β (134, 135).

Furthermore, dysbiosis of the GM also instigate the migration of autoreactive cells to the joints, leading to local inflammation and damage (136). *Collinsella aerofaciens* increases intestinal permeability and worsens arthritis by reducing TJ protein expression (15). In contrast, *Faecalibacterium prausnitzii* preserves intestinal barrier integrity, maintain the balance between Th17 and Treg cells, and exhibit substantial anti-inflammatory effects (137). Loss of beneficial bacteria like Akkermansia muciniphila also impairs epithelial barrier function; its protein Amuc_1000 enhances Claudin-3 and Occludin via TLR2 signaling (138). It is worth noting that *A. muciniphila* is classified as a mucin-degrading bacterium, which can influence the integrity of the mucin barrier (139, 140). These findings suggest that alterations in gut microbiota diversity may impair intestinal mucosal permeability, thereby facilitating the onset of RA (26, 141).

Microbial metabolites function as exogenous regulators of the TJ barrier. For example, butyrate enhances the expression of Cldn1 (encoding Claudin-1) and Ocln (encoding occludin) via hypoxia-inducible factor 1 (HIF-1), conferring resistance to barrier disruption and bacterial translocation following *Clostridium* difficile infection (142). In intestinal epithelial cells, indole-3-propionic acid down-regulates TNF-α and up-regulates TJ-related proteins through pregnane X receptor (PXR) signaling (143). Urolithin A, derived from polyphenols, modulates TJs through AhR signaling (144). *Lactobacillus* species generate hydroxy fatty acids like 10-hydroxy-cis-12-octadecenoic acid (HYA), which activates MAPK/extracellular signal-regulated kinase (ERK) signaling and upregulates TJ-related proteins (145, 146).

Collectively, these findings suggest that specific symbionts influence epithelial barrier function through the provision of beneficial metabolites and proteins.

### 3.5 The GM derived molecular mimicry of autoantigens

Molecular mimicry is a mechanism implicated in the pathogenesis of RA, characterized by the structural similarities between bacterial peptides and host antigens or receptors, leading to immune cross-reactivity and autoimmunity. GM produces metabolites resembling host molecules, and peptides from species like Firmicutes and Proteobacteria show homology with human proteins such as N-Acetyl-glucosamine-6-sulfatase (GNS) and filamin A (FLNA), which are targeted in RA (147, 148). HLA-DR-presented GNS and FLNA peptides also exhibit sequence homology with bacterial epitopes from *Prevotella* sp., *Parabacteroides* sp., and *Butyricimonas* sp. (148). Additionally, shared sequences between *Collinsella* and DRB1*0401 suggest that *Collinsella* may induce RA through molecular mimicry (15). These findings provide evidence for molecular mimicry as a potential mechanism linking disrupted mucosal immune tolerance and systemic immunity in RA patients.

In summary, the gut-immune axis in RA underscores the interplay between dysbiosis, metabolite dysregulation, barrier defects, and autoantigen mimicry. Future therapies aimed at modulating GM or their metabolites hold promise for restoring immune equilibrium and halting RA progression.

## 4 TCM therapy via modulating the gut-immune axis

TCM therapy targets the gut-immune axis for RA through multiple mechanisms, including modulating microbial composition, regulating GM-derived metabolites, and enhancing intestinal barrier function

### 4.1 TCM formulas

**Table 1** summarizes TCM formulas that demonstrate anti-RA activities through modulation of gut–immune axis.

**Table 1:** The effects and mechanisms of anti-RA TCM formulas on the gut–immune axis.

TCM formulas	Main Ingredients	GM Modulation (↓↑)	Effects and mechanisms	References
Wu-tou decoction	*Aconitum carmichaelii* Debeaux, *Ephedra sinica* Stapf, *Astragalus membranaceus* (Fisch.) Bunge, *Paeonia lactiflora* Pall., and Radix Glycyrrhizae Preparata	↓ *Akkermansia*, *Prevotella*, *Bacteroides* ↑ *Oscillospira*, *Lactobacillus*	↓Inflammation, TNF-α, IL-1β, MCP-1, MMP-3, NF-κB/p38; ↑ PPARγ; ↓ CD4^+^/CD8^+^ T cell ratio, ↑ M2 macrophage polarization, ↑ SCFAs, lactate, IAA, IPA, IAld, activate AhR, ↑ gut barrier	(34, 149–152; (153, 154)
Qing-re-huo-xue decoction	*Smilax glabra* Roxb., *Lonicera japonica* Thunb., *Atractylodes lancea* (Thunb.) DC., stir-fried, *Phyllodendron chinense* C.K.Schneid., *Paeonia veitchii* Lynch, *Medicago sativa* L., Salvia miltiorrhiza Bunge, *Curcuma phaeocaulis* Valeton, *Sinomenii Caulis*, *Scolopendra subspinipes mutilans*, Nidus Vespae	↑ Increased the abundance and species evenness of GM	↑Treg cells, ↓Th17 cells, rebalances the Th17/Treg axis, anti-inflammatory immune regulation	(29, 44, 155)
Dang-gui-nian-tong Decoction	*Notopterygium incisum* K.C. Ting ex H.T. Chang, Atractylodes *macrocephala* Koidz., Artemisia capillaris Thunb., *Panax ginseng* C. A. Mey., Radix Glycyrrhizae Preparata, *Sophora flavescens* Aiton, *Angelica sinensis* (Oliv.) Diels, *Actaea cimicifuga* L., *Polyporus umbellatus (Pers.)* Fries, *Puerariae Lobatae* Radix, *Scutellaria baicalensis* Georgi, *Atractylodes lancea* (Thunb.) DC., *Alisma plantago-aquatica* L., *Anemarrhena asphodeloides* Bunge, *Saposhnikovia divaricata* (Turcz.) Schischk.	↑*Lactobacillus*, *Prevotella*, and *Alloprevotella* ↓*Bacteroides*	↓ The hyperplasia and inflammation of synovial tissue; ↓the arthritis index(AI)	(156–160)
Jin-wu-jian-gu Capsules	*Cibotium barometz* (Linn.) J. Sm., *Periploca forrestii* Schltr., *Sabia parviflora* Wall. ex Roxb., *Homalomena occulta* (Lour.) Schott, *Curcuma longa* L., *Zaocys dhumnade*, *Panax notoginseng* (Burkill) F. H. Chen ex C. Y. Wu & K. M. Feng, *Radix Paeoniae Alba*, and *Glycyrrhiza uralensis* Fisch.	↑ *Lachnospira Bryantii*, *Small_NK4A136_group*; ↓ *Prevotella Shan & Collins*, *Helicobacter*	↓Immune response, inflammation, ↓ pro-inflammatory cytokines, IL-1β & IL-18, NLRP3/Caspase-1, IL-33/ST2 binding; ↓ Pyroptosis	(14, 161, 162; (74, 163, 164)
Li-jie Capsule	*Astragalus membranaceus* (Fisch.) Bunge, *Atractylodes lancea* (Thunb.) DC., *Arisaema cum* Bile, *Coix lacryma-jobi* L., *Angelicae pubescentis* radix, *Paeonia veitchii* Lynch, *Ligusticum chuanxiong* Hort, Atractylodes *macrocephala* Koidz., *Pericarpium Citri Reticulatae*, *Gentiana macrophylla* Pall., *Lonicerae Japonicae Caulis*, *Rehmannia glutinosa* Libosch, *Anemarrhena asphodeloides* Bunge, *Angelica dahurica* (Fisch. ex Hoffm.) Benth. & Hook. f. ex Franch. & Sav., *Saposhnikovia divaricata* (Turcz.) Schischk., *Glycyrrhiza uralensis* Fisch.	↑*Barnesiella, Bifidobacterium, Allobaculum*, and *Erysipelotrichace* ↓*Desulfovibrio, Streptococcus*, and *Clostridium XlVa*	↑CD3+, CD8+ cell counts ; ↓the CD4+/CD8+ ratio	(165, 166)
New-bi-tong-ling	*Cinnamomi Ramulus*, *Sinomenii Caulis*, *Saposhnikovia divaricata* (Turcz.) Schischk., *Aconiti radix*, Ephedrae herba, and Nidus Vespae	↑Mycoplasma taceae, *Metamycoplasma_sualvi.* ↓*Prevotellaceae* *_Ga6A1_group*	↓Inflammatory Cytokines (TNF- α, IL-17, IL-6); ↓VEGF, VEGFR1, VEGFR2, HIF-1α; ↑miR-20a-5p, miR-223-3p	(68, 167, 168)
Zhu-bi decoction	*Curculigo orchioides* Gaertn., *Epimedium brevicornu* Maxim., *Morinda officinalis* How, *Angelica sinensis* (Oliv.) Diels, *Anemarrhena asphodeloides* Bunge, *Cortex Phellodendri* Chinensis, *Buthus martensii Karsch*, and *Scolopendra subspinipes mutilans*	↑Firmicutes*, Clostridia, Bacilli.* ↓*Prevotella_9, Ligilactobacillus*, *Prevotellaceae and Tuzzerella*	Restore GM diversity, balance metabolic and immune pathways (PI3K/AKT)	(169–171)
Jing-fang Granule	*Schizonepeta tenuifolia* (Benth.) Briq., *Notopterygium incisum* K.C. Ting ex H.T. Chang, *Saposhnikovia divaricata* (Turcz.) Schischk., *Heracleum hemsleyanum* Diels, *Bupleurum chinense* DC., *Ligusticum striatum* DC., *Citrus aurantium* L., *Poria cocos* (Schw.) Wolf., *Peucedanum praeruptorum* Dunn, *Platycodon grandiflorus* (Jacq.) A.DC., and *Glycyrrhiza uralensis* Fisch.	↑Bacteroidota, *Norank_f_Muribaculaceae, Butyricicoccus, Adlercreutzia* and *Enterorhabdus*; ↓Firmicutes and *Lactobacillus*	↓ TNF-α, IL-1β, IL-6, NLRP3, TLR4/NF-κB pathways, lipid oxidative stress-induced ferroptosis,↑AMPK signaling, Claudin 5 and ZO-1	(42, 172, 173)

↑=Increase/Promote/Upregulate, ↓=Decrease/Inhibit/downregulate.

#### 4.1.1 Wu-tou decoction

WTD, a classical TCM formula, was originally recorded in the “Jin Kui Yao Lue” by the renowned Chinese medical sage Zhang Zhongjing. This decoction is composed of five primary herbs: *Aconitum carmichaelii* Debeaux, *Ephedra sinica* Stapf, *Astragalus membranaceus* (Fisch.) Bunge, *Paeonia lactiflora* Pall., and Radix Glycyrrhizae Preparata. It is widely manufactured in China following the quality control standards set by the Chinese Pharmacopoeia. Clinically, WTD has been extensively applied for treating conditions such as RA, constitutional hypotension, and hemicrania (149, 150). Compared with the MTX, WTD significantly decreased the 28-joint disease activity score (DAS28) and the levels of TNF-α and IL-6 in RA patients with cold-damp syndrome, furthermore, it can improve clinical symptoms and significantly reduce the serum levels of pro-inflammatory cytokines in RA patients (34).

WTD effectively alleviates arthritis in adjuvant-induced arthritis (AIA) rats by modulating GM composition. Specifically, WTD significantly reduces the abundance of *Akkermansia*, *Prevotella*, *Bacteroides*, *Enterococcus*, *Dorea*, and *Jeotgalicoccus*, while increasing *Oscillospira* and *Lactobacillus* populations. Correlation analysis further reveals that WTD’s therapeutic effects are partially mediated by up-regulating microbial metabolites, including SCFAs, lactate, and tryptophan derivatives (indole-3-acetic acid/IAA, indole-3-propionic acid/IPA, and indole-3-aldehyde/IAld), which collectively regulate inflammatory responses and enhance intestinal barrier function, furthermore, IAA, IPA, and IAld possess anti-inflammatory properties and can serve as ligands for the AhR. The activation of AhR can modulate innate and adaptive immune responses in a ligand-specific manner (151).

WTD significantly decreased the expression of TNF-α, IL-1β, monocyte chemoattractant protein-1 (MCP-1), and MMP-3 in the synovium, mitigating arthritis. WTD suppressed M1-type macrophage polarization while promoting M2-type polarization both *in vitro* and *in vivo*. Additionally, WTD inhibited NF-κB and p38 phosphorylation in CIA rats and LPS-induced RAW264.7 macrophages, enhanced PPARγ nuclear translocation, and consequently alleviated synovial inflammation (152). It regulates immune responses by altering CD4+/CD8+ ratios in the AIA rats (153). The five constituent herbs in WTD have synergistic anti-arthritic effects on RA. Radix Aconite is the main anti-inflammatory component. Herba Ephedrae inhibits NF-κB mediated inflammation. Radix Astragali enhances the NF-E2-related factor 2 (Nrf2) expression. Collectively, WTD inhibits NF-κB phosphorylation and increases Nrf2 expression (154). These findings suggest WTD as a promising microbiota-targeted therapy for RA.

#### 4.1.2 Qing-re-huo-xue decoction

QRHXD is made up of eleven TCMs: *Smilax glabra* Roxb., *Lonicera japonica* Thunb., *Atractylodes lancea* (Thunb.) DC., stir-fried, *Phyllodendron chinense* C.K.Schneid., *Paeonia veitchii* Lynch, *Medicago sativa* L., Salvia miltiorrhiza Bunge, *Curcuma phaeocaulis* Valeton, *Sinomenii Caulis*, *Scolopendra subspinipes mutilans* Nidus Vespae. A five-year radiological study demonstrated that QRHXD exhibits a significant therapeutic effect on RA patients, primarily by slowing the long-term progression of bone destruction (29). A multicenter, double-blind, randomized controlled trial (RCT) demonstrated that QRHXD was effective in alleviating symptoms of active RA, although its efficacy was slightly lower compared to csDMARDs. Notably, QRHXD has fewer side effects (44). In a rat CIA model, QRHXD significantly alleviated pathological lesions in synovium and cartilage, increased the abundance and species evenness of GM, elevated Treg levels, and concurrently reduced Th17 levels. These findings suggest that QRHXD may alleviate RA symptoms by improving intestinal microecological imbalance and modulating the immune dysregulation of the Th17/Treg axis (155).

#### 4.1.3 Dang-gui-nian-tong decoction

DGNTD, a well-established TCM formula, is widely acknowledged for its efficacy in alleviating dampness and treating RA. Originating from the Qing Dynasty, DGNTD is currently listed in the National Health Insurance Directory of China (174). This decoction comprises fifteen distinct TCMs, including *Notopterygium incisum* K.C. Ting ex H.T. Chang, Atractylodes *macrocephala* Koidz., Artemisia capillaris Thunb., *Panax ginseng* C. A. Mey., Radix Glycyrrhizae Preparata, *Sophora flavescens* Aiton, *Angelica sinensis* (Oliv.) Diels, *Actaea cimicifuga* L., *Polyporus umbellatus (Pers.)* Fries, *Puerariae Lobatae* Radix, *Scutellaria baicalensis* Georgi, *Atractylodes lancea* (Thunb.) DC., *Alisma plantago-aquatica* L., *Anemarrhena asphodeloides* Bunge, *Saposhnikovia divaricata* (Turcz.) Schischk. Previous clinical study indicated DGNTD has good therapeutic effects on early RA patients with damp-heat obstruction syndrome (156, 157). DGNTD effectively mitigates the hyperplasia and inflammation of synovial tissue in AIA model rats, thereby inhibiting pannus formation. DGNTD increased the abundance of *Lactobacillus*, *Prevotella 9*, and *Alloprevotella*, while reducing the abundance of *Bacteroides*. *Bacteroides* and *Helicobacter* positively correlated with the arthritis index (AI), while *Prevotella 9* and *Candidatus Saccharimonas* negatively correlated with AI. *Prevotella 9* abundance showed significant negative correlations with paw volume and spleen index (158), whereas Ruminococcaceae_NK4A214_group, Christensenellaceae_R-7_group, and *Bacteroides* were positively associated with spleen index. Ruminococcaceae exhibits pro-inflammatory effects by activating immune cells and stimulating pro-inflammatory cytokine secretion (159), while Christensenellaceae_R-7_group modulates lipid metabolism and SCFA levels, both of which are closely linked to immune regulation (160). The results suggest that these microbial changes may be linked to immune response modulation.

#### 4.1.4 Jin-wu-jian-gu (JWJG)Capsules

JWJG Capsules, a renowned Chinese Miao medicinal formula, is widely recognized for its efficacy in promoting bone repair and treating RA. JWJG Capsule in combination with leflunomide can effectively alleviate joint and systemic symptoms in RA patients with cold-dampness obstruction syndrome, reduce inflammatory markers, demonstrate superior efficacy compared to leflunomide monotherapy, and maintain good safety (161). The formula comprises nine traditional herbs: *Cibotium barometz* (Linn.) J. Sm., *Periploca forrestii* Schltr., *Sabia parviflora* Wall. ex Roxb., *Homalomena occulta* (Lour.) Schott, *Curcuma longa* L., *Zaocys dhumnade*, *Panax notoginseng* (Burkill) F. H. Chen ex C. Y. Wu & K. M. Feng, *Radix Paeoniae Alba*, and *Glycyrrhiza uralensis* Fisch. JWJG-medicated serum significantly suppresses the expression of Nod-like receptor pyrin domain-containing 3(NLRP3) and caspase in RA synovial fibroblasts (SF), inhibiting the maturation of IL-1β and IL-18, mitigating pyroptosis (162). JWJG also modulates immune-inflammatory responses by down-regulating pro-inflammatory cytokines, including TNF-α, IL-6, IL-13, IL-17, and IL-1β, as well as by inhibiting inflammatory cell infiltration. Liquiritigenin, identified as the key component through network pharmacology, inhibits the IL-33/Suppression of Tumorigenicity 2 (ST2) receptor complex, reducing inflammation (163). JWJG capsules significantly altered the GM composition in CIA model rats, specifically up-regulating *Lachnospira* Bryant & Small_NK4A136_group while down-regulating the relative abundances of *Prevotella* Shan & Collins and *Helicobacter* Gest & Favinger (93, 175). Clinical studies show higher *Prevotella* levels in untreated RA patients, suggesting its role in disease development (14), while *Lachnospira* may be beneficial. Notably, JWJG capsules reduced *Prevotella* abundance in CIA rats, further supporting its therapeutic effect through microbiota regulation. Collectively, these combined actions on molecular inflammatory mechanisms and gut dysbiosis underpins JWJG’s effectiveness in alleviating RA symptoms and pathology.

#### 4.1.5 Li-jie Capsule

*Li-jie Capsule* has been used in the treatment of RA for many years because of its better therapeutic effects and lower incidence of side effects (96). The main ingredients of Li-jie Capsule are *Astragalus membranaceus* (Fisch.) Bunge, *Atractylodes lancea* (Thunb.) DC., *Arisaema cum* Bile, *Coix lacryma-jobi* L., *Angelica sinensis* (Oliv.) Diels, *Paeonia veitchii* Lynch (Chi Shao), *Ligusticum chuanxiong* Hort, Atractylodes *macrocephala* Koidz. (Bai Zhu), *Pericarpium Citri Reticulatae*, *Gentiana macrophylla* Pall., *Lonicerae Japonicae Caulis*, *Rehmannia glutinosa* Libosch, *Anemarrhena asphodeloides* Bunge, *Angelica dahurica* (Fisch. ex Hoffm.) Benth. & Hook. f. ex Franch. & Sav., *Saposhnikovia divaricata* (Turcz.) Schischk., *Glycyrrhiza uralensis* Fisch. The Li-jie Capsule alleviates joint symptoms, improves joint function, and modulates immunity in RA patients by increasing CD3+ and CD8+ cells, lowering the CD4+/CD8+ ratio, and reducing erythrocyte sedimentation rate (ESR) and RF levels. This indicates a reduction in humoral immune response and an enhancement of cellular immune response, thereby exerting immunomodulatory effects. It shows better systemic symptom improvement and cellular immune regulation than Tripterygium glycosides Tablets. The comprehensive therapeutic effect of the Li-jie Capsule on RA may be attributed to its modulation of T cell immune function (165). Li-jie Capsule significantly reduces paw swelling and AI values in CIA mice. Additionally, Li-jie Capsule markedly decreased the levels of *Desulfovibrio*, *Streptococcus*, and *Clostridium XlVa*, while increasing the levels of *Barnesiella*, *Bifidobacterium*, *Allobaculum*, and *Erysipelotrichace*. These findings suggest the Li-jie Capsule exerts therapeutic effects on RA through immune modulation and GM regulation (166).

#### 4.1.6 New-bi-tong-ling

NBTL, a well-established TCM formula, is widely acknowledged for its efficacy in treating RA (176). It is composed of six herbs, including *Cinnamomi Ramulus*, *Sinomenii Caulis*, *Saposhnikovia divaricata* (Turcz.) Schischk., *Aconiti radix*, Ephedrae herba, and Nidus Vespae. NBTL reduces joint swelling, bone destruction, and pro-inflammatory cytokines (IL-1β, IL-6) in CIA rats, while increasing body weight and anti-inflammatory cytokines (IL-10, IL-4). It also inhibits FLS inflammation, induces apoptosis, and hinders proliferation, which was reversed by JAK2/STAT3 activation (167). Another study confirms NBTL alleviates RA by reducing the expression levels of TNF-α, IL-17, IL-6, and apoptosis-associated speck-like protein containing a CARD in synovial tissues. It modulates GM linked to the VEGF pathway, up-regulating f_Mycoplasmataceae and *s_Metamycoplasma_sualvi*, while down-regulating *g_Prevotellaceae_Ga6A1_group*. NBTL suppresses the VEGF signaling pathway and angiogenesis by inhibiting VEGF, its receptors, and HIF-1α. It also up-regulates microRNA-20-5p (miR-20a-5p) and miR-223-3p, reducing angiogenesis, and lowers the CD4+/CD8+ ratio along with IL-2 and IL-2R levels (168, 177). Morphological observation showed inhibitory effects on synovial cell proliferation (68). These findings suggest NBTL has therapeutic potential in RA by regulating microbiota and the VEGF pathway, supporting its promise as a treatment option requiring further study.

#### 4.1.7 Zhu-bi decoction

ZBD has been utilized for many years in RA treatment. Originating from the classical TCM prescription “Erxian decoction” which is recorded in “the Clinical Manual of Chinese Medical Prescriptions”, it has been demonstrated to be effective in treating RA, with minimal side effects (169, 170). This prescription has since been modified to meet modern clinical needs while preserving its therapeutic efficacy. ZBD consists of eight distinct herbs, specifically *Curculigo orchioides* Gaertn., *Epimedium brevicornu* Maxim., *Morinda officinalis* How, *Angelica sinensis* (Oliv.) Diels, *Anemarrhena asphodeloides* Bunge, *Phellodendron Chinense* C.K.schneid., *Buthus martensii Karsch*, and *Scolopendra subspinipes mutilans*. ZBD effectively alleviates RA symptoms in CIA rats without significant side effects, showing efficacy comparable to that of MTX. It mitigates inflammation and joint damage by modulating the phosphatidylinositol 3-kinase (PI3K)/protein kinase B (PKB/AKT) (PI3K/AKT) signaling pathway and reducing serum concentrations of cytokines, including TNF-α, IL-1β, and IL-6. ZBD modulates 170 differential metabolites and partially restores disrupted metabolic profiles. It also mitigates gut dysbiosis and identifies key bacterial genera associated with the treatment effects. Specifically, it increases Firmicutes, Clostridia, and Bacilli abundance while reducing *Prevotella_9*, *Ligilactobacillus*, Prevotellaceae, and *Tuzzerella*. In conclusion, ZBD alleviated RA by restoring GM diversity and balancing metabolic and immune pathways, and was a safe and efficacious TCM formula for treating RA (171).

#### 4.1.8 Jing-fang Granule

JFG is a modern formula derived from Jing-fang-Bai-du Powder, a traditional prescription originating from the Ming Dynasty. It retains the same herbal composition and dosage as its predecessor. JFG comprises 11 herbal medicines: *Schizonepeta tenuifolia* (Benth.) Briq., *Notopterygium incisum* K.C. Ting ex H.T. Chang, *Saposhnikovia divaricata* (Turcz.) Schischk., *Heracleum hemsleyanum* Diels, *Bupleurum chinense* DC., *Ligusticum striatum* DC., *Citrus aurantium* L., *Poria cocos* (Schw.) Wolf., *Peucedanum praeruptorum* Dunn, *Platycodon grandiflorus* (Jacq.) A.DC., and *Glycyrrhiza uralensis* Fisch. Over an extended period, JFG has been widely applied in the treatment of inflammatory diseases, including RA (42, 172). JFG protects rats from RA by reducing foot swelling, improving synovial pathology, and lowering TNF-α, IL-1β, and IL-6 levels via NLRP3 and TLR4/NF-κB inhibition. It reshapes GM by enhancing Bacteroidota, *Butyricicoccus*, *Adlercreutzia* and *Enterorhabdus* while decreasing Firmicutes and *Lactobacillus*. This leads to higher levels of acetic, propionic, and butyric acids in the gut and serum. These changes activate AMPK signaling, which regulates fatty acid metabolism and biosynthesis, thereby inhibiting lipid oxidative stress-induced ferroptosis and alleviating tissue damage associated with RA. JFG also strengthens the intestinal barrier by upregulating Claudin 5 and ZO-1 (173). This research provides a new mechanism for JFG’s effect on RA through the “Gut-joint” axis.

### 4.2 Single TCM and its components

#### 4.2.1 Tripterygium wilfordii Hook F

TwHF is a traditional medicinal Chinese herb which has been extensively utilized for a long period in the treatment of various autoimmune disorders and inflammatory diseases, including RA (178, 179). Increasing studies have indicated that TwHF might represent a rich source that possesses multiple pharmacological activities, particularly anti‐inflammatory, anticancer, antiviral, and antioxidative activities (180). The efficacy and safety of TwHF have been substantiated through multiple multi-center RCTs. A multi-center, open-label RCT demonstrated that TwHF monotherapy was non-inferior to MTX monotherapy, while the combination of MTX and TwHF was superior in controlling disease activity in RA patients (180). A systematic review of data up to 2016 further revealed that TwHF was more effective in improving the American College of Rheumatology (ACR)20 and ACR50 response rates compared to DMARDs. However, TwHF has been associated with adverse menstrual effects (37).

Tripterygium glycosides (TG) are the active components derived from Celastraceae *Tripterygium wilfordii* Hook. F. (TwHF), which encompass a variety of diterpenoids, alkaloids, triterpenoids, and glycosides (181). TG regulates multiple signaling pathways and inflammatory factors in RA patients, including upregulating alpha7 nicotinic acetylcholine receptor (α7nAChR) expression, inhibiting NF-κB and STAT3 activation, and reducing IL-17 and high mobility group box protein 1 (HMGB1) levels (182).TG tablets (TGTs) combined with MTX significantly improve RA symptoms and immune function by increasing CD3+ and CD4+/CD8+ T lymphocyte levels in RA patients (41, 178, 183). TGTs reduced joint swelling and lowered IL-6 and TNF-α in CIA rats. TGTs significantly down-regulated the abundances of *Akkermansia*, *Prevotellaceae_NK3B31_group*, and notably, *Prevotella*, which is closely associated with RA in CIA rats. Conversely, TGTs significantly increased the abundances of *Ureibacillus*, *Lactobacillus*, *Butyricicoccus*, and *Ruminococcus_UCG-014*. Additionally, after TGTs treatment, the levels of *Blautia*, which is related to inflammation, as well as *Escherichia-Shigella* and *Lachnoclostridium*, returned to levels comparable to those observed in normal rats (184). These mechanisms suggest that TG may alleviate RA by enriching butyrate-producing microbiota, reducing *Prevotella*, and suppressing inflammatory pathways (NF-κB/STAT3) and cytokines (IL-6, TNF-α, IL-17).

#### 4.2.2 Radix Paeoniae Alba

*Radix Paeoniae Alba* is a constituent of JWJG Capsules. Total glucosides of paeony (TGP), an extract from the dried root of *Radix Paeoniae Alba*, contain bioactive compounds such as paeoniflorin, hydroxypaeoniflorin, and paeonin. These compounds exhibit anti-inflammatory, immunomodulatory, antithrombotic, and hepatoprotective properties. TGP can inhibit autoimmune reactions and maintain immune tolerance in the body through multiple pathways. As an adjuvant therapy, TGP has demonstrated efficacy in managing autoimmune diseases, including systemic lupus erythematosus, Sjogren’s syndrome, RA, ankylosing spondylitis, and immune-related recurrent abortions. Furthermore, TGP treatment can reduce adverse drug reactions, lower recurrence rates, and enhance patient compliance (185). The results of a systematic review of 1,209 patients with active RA showed that, compared to no additional treatment, the addition of TGP to traditional DMARD(s) may significantly improve ACR 20, ACR 50, and ACR 70 response rates, as well as reduce adverse effects (46). Therefore, TGP could serve as a promising adjuvant therapy for RA.

TGP administration for 12 weeks corrected 78% of taxonomic differences and significantly increased the abundance of beneficial symbiotic bacteria *Ruminococcaceae_UCG-014*, *Oscillibacter*, and *Parabacteroides*. Additionally, it reduced body weight, thymus index, and inflammatory cell infiltration in the ankle joints of CIA rats. TGP down-regulated VEGF, Th1, and Th17 cells while up-regulating Th2 and Treg cells in CIA rats. Furthermore, TGP administration inhibited the levels of intestinal cytokines, secretory immunoglobulin A (SIgA), and IFN-γ. These findings suggest that the therapeutic effects of TGP may be mediated through gut microbiome regulation and modulation of the intestinal mucosal immune response (186).

#### 4.2.3 Caulis Sinomenii

*Caulis Sinomenii*, a pivotal herb in TCM, is a core component of formulas such as QRHXD, JWJG Capsules, and the patented drug Zheng-qing-feng-tong-ning (ZQFTN). Approved by the China Food and Drug Administration two decades ago for RA, ZQFTN was recently added to China’s National Health Insurance Directory, reflecting its high clinical efficacy and favorable safety profile in RA management (187). Central to its therapeutic action is sinomenine (SIN), a bioactive alkaloid from *Caulis Sinomenii* and an officially recognized RA treatment.

SIN reduces RA disease activity and DAS28 scores by suppressing pro-inflammatory cytokines (e.g., IL-6, TNF-α, IL-1β) and modulates immune cells, including synovial macrophages (CD11b+F4/80+CD64+) and splenic/draining lymph node macrophages (CD11b+Ly6C+CD43+), while lowering CD14+CD16+ monocytes in RA patients. These dual mechanisms—cytokine regulation and immune cell subset modulation—position SIN as a cost-effective alternative or adjunct to methotrexate (MTX) (188). It selectively inhibits microsomal prostaglandin E synthase-1 (mPGES-1), reducing prostaglandin E2 (PGE2) without disrupting prostacyclin (PGI2) or thromboxane A2 (TXA2), potentially minimizing cardiovascular risks compared to NSAIDs. This inhibition is mediated by suppressing NF-κB DNA binding activity (189). Furthermore, SIN mimics MTX by restoring the balance between MMP and tissue inhibitors of matrix metalloproteinase (TIMP), protecting bone integrity and acting as a natural DMARDs to slow RA (190). SIN enriches anti-CIA *Lactobacillus* species (*L. paracasei* and *L. casei*) and boosts microbial tryptophan metabolites (indole-3-acrylic acid, indole-3-propionic acid, and indole-3-acetic acid), which activate the AhR. AhR activation rebalances Th17/Treg cells, alleviating arthritis severity in preclinical models. Mono-colonization studies confirm that these *Lactobacillus* strains contribute directly to SIN’s efficacy, underscoring a “microbiota-metabolite-immunity” axis as a core mechanism (191).

As a multifaceted agent, SIN combines immunosuppressive, anti-inflammatory, and microbiota-modulating properties, offering a holistic approach to RA treatment. Its ability to target both inflammatory pathways and gut dysbiosis highlights its potential as a novel therapeutic strategy.

#### 4.2.4 Phyllodendron chinense C.K.Schneid

The utilization of *Phyllodendron chinense* C.K.Schneid. in the aforementioned TCM formula for treating RA has been documented (29). Berberine (BBR), an isoquinoline alkaloid derivative, is one of the primary active components of *Phyllodendron chinense* C.K.Schneid. (192). Research has demonstrated that Berberine exerted an anti-arthritis effect by modulating the GM in CIA rats. Berberine intervention specifically up-regulated butyrate-producing genera positively correlated with anti-inflammatory effects, including *Blautia*, *Butyricicoccus*, and *Parabacteroides*, while down-regulating butyrate-suppressing genera linked to pro-inflammatory responses such as *Prevotella*, *Paraprevotella*, and *Coprococcus*. Mechanistically, berberine reduced splenic levels of pro-inflammatory cytokines, particularly Th17-associated IL-17A, IL-17F, IL-21, and IL-22, through suppression of RORγt expression and STAT3 phosphorylation. Crucially, antibiotic treatment abolished these immunomodulatory effects, collectively demonstrating berberine’s microbiota-dependent therapeutic potential in RA (70).

#### 4.2.5 Daphne giraldii Nitsche

The root bark and stem bark of *Daphne giraldii* Nitsche, a plant of the genus Daphne in the Thymelaeaceae family, are known as Zushima. The main active components are daphnetin and zushima saponin (193). The Zushima tablet (ZT) has a wide therapeutic basis in Chinese folk medicine and is often used to treat conditions such as pain, injuries from falls, and RA. Clinical observations have shown that the curative effect of ZT in the treatment of RA is better than that of ZQFTN Tablets (194, 195). The study demonstrated that ZT effectively ameliorated CIA. 16S rRNA analysis revealed Firmicutes and Bacteroidetes as the dominant bacterial phyla in the GM of CIA rats. At the family level, 19 bacterial taxa were significantly altered in RA-model rats. Fecal metabolomics further indicated that ZT up-regulated propionate, butyrate, and valerate levels in CIA rats, with the therapeutic mechanism potentially linked to SCFAs enhancing disease mitigation through increased Treg populations (196). Therefore, the therapeutic mechanism of ZT involves the gut microbiome-driven immunomodulation and solidifies its role as a potent RA treatment.

#### 4.2.6 Panax ginseng C. A. Mey. (P. ginseng)

*P. ginseng* first documented in the “Shen Nong Materia Medica”, is one of the principal components in DGNTD (174). *P. ginseng* contains a variety of bioactive compounds, including ginsenosides, polysaccharides, amino acids, and others, with ginsenosides being the primary active constituents (197). Research has demonstrated that ginsenoside Rg2, a triol-type saponin, enhances intestinal colonization of *Parabacteroides distasonis*, which directly suppresses Th17 cell differentiation through the production of bioactive metabolites-LCA, DCA, isoLCA, and 3-oxoLCA. Specifically, 3-oxoLCA and isoLCA not only directly suppressed the differentiation of Th17 cells but were also recognized as TGR5 agonists, enhancing the M2 polarization of macrophages. These dual mechanisms-microbiota-dependent immunomodulation and macrophage reprogramming-were validated in both CIA mice and TNF-Tg murine models (129). Therefore, as a prebiotic agent, Rg2 exerts therapeutic effects on arthritic mice by promoting the proliferation of *P. distasonis*.

#### 4.2.7 Clematis chinensis Osbeck

*Clematis chinensis* Osbeck is a key component of Wang-Bi Tablet (WB), which has been patented in China and widely used for the treatment of RA due to its excellent therapeutic efficacy and minimal side effects (43). A study showed that both crude extracts and wine-processed *Clematis chinensis* Osbeck increase Firmicutes and decrease Bacteroidetes, while reducing *Prevotella*, *Bacteroides*, and *Blautia* and increasing *Paraprevotella* in the model group (198). In addition, the extract of C. chinensis can inhibit NO produced by peritoneal macrophages, which indicated that C. chinensis methanol extract had an obvious immunosuppressive effect (199). This combination of GM restoration and immunomodulation underpins its therapeutic value in RA treatment and supports its use in WB.

#### 4.2.8 Toddalia asiatica (L.) Lam.

*Toddalia asiatica* (L.) Lam. is contained in Ba-wei-long-zuan granule (BLG), a traditional Chinese Zhuang medicine used for treating RA (43). A recent study has indicated that the extract of *Toddalia asiatica* (L.) Lam. (TAE) alleviates joint symptoms in rats with RA by restoring the balance of Th17/Treg cells in the colon and rectifying gut dysbiosis. TAE downregulated the expression levels of IL-17A, IL-1β, and IL-6 in the colon while up-regulating FOXP3 and IL-10, indicating its regulatory role in the intestinal Th17/Treg balance. Furthermore, TAE improved GM diversity in AIA rats, reducing the abundance of *Ligilactobacillus*, which was elevated in the model group, and increasing the relative abundance of *Muribaculum*, *Subdoligranulum*, *Lachnospira*, and *Marvinbryantia(* 200 *)*. These findings provide evidence that the efficacy of *Toddalia asiatica* (L.) Lam. and its inclusion in BLG for RA involves a dual mechanism targeting the gut-joint axis: immunomodulation and GM restoration.

#### 4.2.9 Glycyrrhiza uralensis Fisch. (G. uralensis)

*G. uralensis* is one component of JWJG Capsules, used for treating RA and showing potent anti-inflammatory activity (43). *G. uralensis* treatment significantly improves joint inflammation, pathological lesions, and inflammation markers in CIA rats. It reverses abnormal GM composition by increasing *Eubacterium*, *Roseburia*, *Desulfovibrio*, *Bacteroides*, *Ruminococcaceae_Clostridium*, and *Peptostreptococcaceae_Clostridium*, while reducing *Helicobacter*, *Prevotella*, *Lachnospiraceae_Clostridium*, and *Barnesiella*. Meanwhile, *G. uralensis* alleviates intestinal damage, enhances intestinal barrier integrity by upregulating TJ proteins (ZO-1, occludin, and claudin-1). It also lowers Th17/Treg cell ratios in blood, colon, and joint fluid. These effects suggest that G. uralensis alleviates RA symptoms by modulating GM and immunity (201).

#### 4.2.10 Notopterygium incisum K.C. Ting ex H.T. Chang (N. incisum)

*N. incisum* is a key component of DGNTD and JFG (174). Polysaccharides derived from *N. incisum* may represent one of its primary active constituents. A novel polysaccharide, named NIP, was isolated from *N. incisum* with a molecular weight of 2.34×10 ^6^ Da. NIP consists of arabinose, galactose, glucose, and galacturonic acid, linked by methyl esterified 1,4-linked α-galacturonic acid, 1,6-linked β-galactose, 1,5-linked α-arabinose, and 1,4,6-linked β-glucose. NIP suppresses NO production in LPS-stimulated RAW264.7 macrophages. NIP reduces toe inflammation in AIA rats, suppresses inflammatory cytokine release, and inhibits NF-κB and JAK/STAT3 pathway activation. Furthermore, NIP mitigated oxidative stress by decreasing malondialdehyde (MDA) levels and enhancing superoxide dismutase (SOD) activity in a dose-dependent manner. Additionally, NIP significantly decreases thymus and spleen indices, indicating immunosuppressive effects. NIP also markedly increases GM diversity, restores the Bacteroidetes-to-Firmicutes ratio, a critical index associated with disease susceptibility. Moreover, NIP enhances the abundance of *Eisenbergiella*, a genus known for producing butyrate, an anti-inflammatory metabolite (202, 203). These findings suggest NIP exerts anti-RA effects through anti-inflammatory, antioxidant, and GM-modulating mechanisms (204).

### 4.3 Herb couple

*Angelica sinensis* (Oliv.) Diels and *N. incisum* are two main constituents of DGNTD (157). The optimal ratio of AN7:3 herb couple was identified, with the active ingredients combination (AIC) screened as key components. AIC showed similar therapeutic effects as AN7:3 in CIA rats and may alleviate RA by regulating the MAPK signaling pathway, metabolic disorders, and gut microbiome-related autoimmunity. This study provides scientific evidence for using AIC as a prebiotic agent for RA and offers a systematic strategy to optimize medicinal material proportions and screen active ingredients in traditional Chinese herb couples (205).

The *in vivo* and *in vitro* effects of single TCM and its active compounds on the gut–immune axis in RA are summarized in **Table 2**.

**Table 2:** The effects and mechanisms of single TCM and its active compounds on the gut–immune axis in RA.

Single TCM	Active compounds	GM Modulation (↓↑)	Effects and mechanisms	Compound’s structure	References
*Tripterygium wilfordii Hook F (TwHF)*	Tripterygium glycosides	↓*Akkermansia*, *Prevotellaceae_NK3B31_group*, *Prevotella.* ↑*Ureibacillus*, *Lactobacillus*, *Butyricicoccus*, *Ruminococcus_UCG-014*	↓ Joint swelling, IL-6, TNF-α, IL-17, HMGB-1; ↑ α7nAChR expression; ↓ NF-κB and p-STAT3 signaling		(37, 178–182, 184, 206)
*Radix Paeoniae Alba*	Total glucosides of paeony (TGP)	↑*Oscillibacter*, *Ruminococcaceae_UCG-014*, *Parabacteroides*	↓Inflammatory infiltration, body weight, thymus index;↓ VEGF, I FN-γ, SIgA; ↑ Th2, Treg, ↓Th1, Th17; Modulate intestinal mucosal immunity		(46, 185, 186)
*Caulis Sinomenii*	Sinomenine (SIN)	↑*Lacticaseibacillus paracasei Lacticaseibacillus casei* *↑* microbial tryptophan metabolites (IA, IPA, IAA)	↓ IL-6, IL-1β, TNF-α, and other cytokines; ↓ mPGES-1 and PGE_2_; ↓ CD11b^+^F4/80^+^CD64^+^ synovial macrophages, CD11b^+^Ly6C^+^CD43^+^ macrophages (spleen, lymph nodes), CD14^+^CD16^+^ monocytes	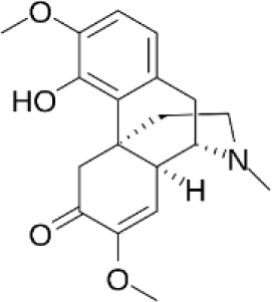	(187–191);
*Phellodendri Chinensis Cortex*	Berberine (BBR)	↓*Prevotella*, *Paraprevotella* and *Coprococcus* ↑*Blautia*, *Butyricicoccus*, *Parabacteroides*	↓ IL-17A, IL-17F, IL-21, IL-22, RORγt expression, STAT3 phosphorylation; Microbiota-dependent immunomodulation	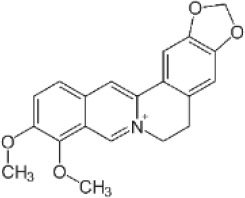	(70, 207)
*Daphne giraldii* Nitsche	Daphnetin, Zushima saponin	Alters 19 gut taxa at the family level; dominant phyla: Firmicutes and Bacteroidetes	↓ inflammation, ↑Treg cells; ↑ SCFAs (propionate, butyrate, valerate)		(193–196)
*Panax ginseng* C. A. Mey.	Ginsenoside Rg2	↑*Parabacteroides distasonis*	↓ Th17 cell differentiation; ↑ M2 macrophage polarization via TGR5 activation; ↑Bile acid metabolites (LCA, DCA, isoLCA, 3-oxoLCA)	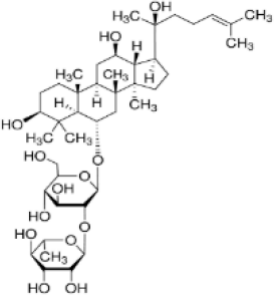	(129, 197)
*Clematis chinensis* Osbeck	Crude extract; Wine-processed extract; Methanol extract	↓ Bacteroidetes*, Prevotella, Bacteroides, Blautia.* ↑ Firmicutes, *Paraprevotella.*	↓ NO production in macrophages; Immunosuppression		(198, 199)
*Toddalia asiatica* (L.) Lam.	Toddalia asiatica (L.) Lam. (TAE)	↓*Ligilactobacillus.* ↑*Muribaculum*, *Subdoligranulum*, *Lachnospira*, *Marvinbryantia.*	↓Levels of IL-17A, IL-1β, and IL-6 in the colon, ↑FOXP3 and IL-10; restore Th17/Treg balance		(200)
*Glycyrrhiza uralensis* Fisch.	*G. uralensis* extract	↓ *Helicobacter, Prevotella, Lachnospiraceae_* *Clostridium, Barnesiella.* ↑ *Eubacterium*, *Roseburia*, *Desulfovibrio*, *Bacteroides*, *Ruminococcaceae_Clostridium*, *Peptostreptococcaceae_Clostridium.*	↓ Th17/Treg ratio in blood, colon, joint fluid; ↑ TJ (ZO-1, occludin, claudin-1), gut barrier		(43, 201)
*Notopterygium incisum* K.C. Ting ex H.T. Chang	*Notopterygium incisum* Polysaccharide (NIP)	↑ *Eisenbergiella.* Restore Bacteroidetes/Firmicutes ratio	↓NF-κB and JAK/STAT3 pathways, NO and cytokines (TNF-α, IL-6). ↓MDA,↑SOD		(202–204)

↑=Increase/Promote/Upregulate, ↓=Decrease/Inhibit/downregulate.

## 5 Conclusions and future prospective

RA, a chronic autoimmune disorder, is intricately linked to dysregulation of the gut-immune axis, where gut dysbiosis, intestinal barrier dysfunction, and immune hyperactivation converge to drive systemic inflammation and joint destruction. Understanding the interactions between GM and the immune system may provide critical insights for developing novel biomarkers and treatment strategies, as well as for elucidating the pathophysiology of RA (**Figures 1**, **2**).

TCM offers a promising therapeutic strategy by targeting this axis through multi-component, multi-pathway mechanisms. TCM can treat RA by improving GM structure, modulating intestinal T lymphocytes, regulating microbiota-derived metabolites, enhancing intestinal barrier function and immunity, and alleviating intestinal dysfunction. TCM not only augments the therapeutic efficacy of conventional RA treatments but also mitigates their side effects. Regulating the gut–immune axis with TCM may become a safer and more effective new method for the treatment of RA, with broad application prospects.

However, there are some current research limitations and model challenges. Interactions between multiple TCM components and the GM are poorly understood. TCM used clinically requires more extensive RCTs to rigorously evaluate efficacy and risks. Due to inherent inconsistencies in TCM formulations, multi-herbal formulas also need greater standardization. This includes addressing variability in plant compounds, batch-to-batch quality, and potential herb-herb interactions, necessitating robust quality control (e.g., HPLC fingerprinting). Widely used AIA/CIA murine models rely on artificial induction, exhibit acute self-limiting inflammation, unlike chronic human RA, and poorly replicate human genetic-environmental interactions (76). Species differences further limit translational relevance.

To overcome these challenges and unlock TCM’s potential, future research should focus on the following aspects: advance disease models. Specifically, prioritize TNF-Tg mice due to their human-like autoimmune and metabolic characteristics, such as chronicity and the gut-joint axis (81). Utilize spontaneous or collagen-induced Nonhuman Primate (NHP) models (e.g., macaques) for high-fidelity TCM trials on pharmacokinetics, toxicity, and microbiota interactions, leveraging their closer immune, metabolic, and genetic resemblance to humans (12). Develop quality controls for TCM compounds to ensure consistency and standardize TCM formulations.

As for deciphering mechanisms, establish the causal role of specific bacterial strains (e.g., *Lactobacillus casei*, *Prevotella copri*) and metabolites (e.g., SCFAs, bile acids) using gnotobiotic models and fecal microbiota transplantation (FMT). Combine metagenomics, metabolomics, and proteomics to map TCM-induced microbial shifts and host pathways, identifying novel biomarkers for personalized therapy. Investigate cross-reactivity between microbial antigens (e.g., *Prevotella*-derived peptides) and host proteins to unravel RA’s autoimmune origins and design targeted interventions. Develop TCM-derived prebiotics and probiotics to reinforce intestinal barrier function and prevent microbial translocation to mitigate RA. Implement stringent quality controls and standardize TCM formulations.

In conclusion, TCM’s ability to harmonize the gut-immune axis offers a transformative, holistic approach to RA treatment. Realizing TCM’s full potential requires resolving mechanistic complexities, advancing clinical validation through rigorous RCTs, and ensuring standardization. Prioritized interdisciplinary collaboration is essential to advance this microbiota-centric approach and improve global RA outcomes.

## Glossary

3-oxoLCA: 3-oxolithocholic acid
ACPAs: anti-citrullinated protein antibodies
ACR: American College of Rheumatology
AhR: aryl hydrocarbon receptor
AI: the arthritis index
APCs: antigen-presenting cells
CIA: collagen-induced arthritis
DAS: joint disease activity score
DAS28: 28-joint disease activity score
DCA: deoxycholic acid
DCs: dendritic cells
DMARDs: disease-modifying anti-rheumatic drugs
ESR: erythrocyte sedimentation rate
FDA: Food and Drug Administration
FLNA: filamin A
FLS: fibroblast-like synoviocytes
FMT: Fecal microbiota transplantation
Foxp3: Forkhead box P3
GCs: glucocorticoids
GF: germ-free
GM: gut microbiota
GNS: N-Acetyl-glucosamine-6-sulfatase
HCs: healthy controls
HDACs: histone deacetylases
HLA: human leukocyte antigen
HLA-DRB1: human leukocyte antigen-DR beta chain 1
IAA: indole-3-acetic acid
IAld: indole-3-aldehyde
IFN: interferon
IL: Interleukin
IPA: indole-3-propionic acid
isoLCA: isolithocholic acid
JAK: Janus kinase
JNK: C-Jun N-terminal kinase
LCA: lithocholic acid
LPD: live P. distasonis
MAPK: Mitogen-activated protein kinase
MCP-1: monocyte chemoattractant protein-1
MDA: malondialdehyde
MHC: major histocompatibility complex
miR-20a-5p: microRNA-20-5p
MLSs: macrophage-like synoviocytes
MMP: Matrix metalloproteinase
mPGES-1: microsomal prostaglandin E synthase 1
MTX: Methotrexate
NETs: neutrophil extracellular traps
NF-κB: Nuclear factor-kappaB
NO: nitric oxide
Nrf2: the NF-E2-related factor 2
NSAID: Non-steroidal anti-inflammatory drug
Ocln: encoding occludin
PADs: peptidyl arginine deiminases
PGE2: Prostaglandin E2
PI3K/AKT: phosphatidylinositol 3-kinase (PI3K)/protein kinase B (PKB/AKT)
PPARγ: peroxisome proliferator-activated receptor gamma
PSA: polysaccharide A
PTMs: Post-translational modiﬁcations
RA: Rheumatoid arthritis
RCT: Randomized controlled trial
RF: rheumatoid factor
ROS: reactive oxygen species
SCFAs: short-chain fatty acids
SFB: Segmented filamentous bacteria
SIgA: secretory immunoglobulin A
SLECs: short-lived effector T cells
SOD: superoxide dismutase
STAT3: signal transducer and activator of transcription 3
TCM: Traditional Chinese Medicine
TCR: T-cell receptor
Tfh: follicular helper T cells
TGF-β: Transforming growth factor β
TGP: Total glucosides of paeony
TGTs: Tripterygium glycosides tablets
Th1: T helper-1 cells
TJ: Tight junction
TLR: Toll-like receptor
TNF: Tumor necrosis factor
Tph: peripheral helper T cells
Tregs: regulatory T cells
TwHF: Tripterygium wilfordii Hook F
TXA2: thromboxane A2
ZO: Zonula Occludens
α7nAChR: alpha7 nicotinic acetylcholine receptor

## References

[1] Weyand CM Goronzy JJ. The immunology of rheumatoid arthritis. Nat Immunol. (2021) 22:10–8. doi: 10.1038/s41590-020-00816-x, PMID: 33257900 PMC8557973

[2] Almutairi K Nossent J Preen D Keen H Inderjeeth C. The global prevalence of rheumatoid arthritis: a meta-analysis based on a systematic review. Rheumatol Int. (2021) 41:863–77. doi: 10.1007/s00296-020-04731-0, PMID: 33175207

[3] D’Orazio A Cirillo AL Greco G Di Ruscio E Latorre M Pisani F. Pathogenesis of rheumatoid arthritis: one year in review 2024. Clin Exp Rheumatol. (2024) 42:1707–13. doi: 10.55563/clinexprheumatol/0307ed, PMID: 39315569

[4] Zhan J Cheng J Chang W Su Y Yue X Wu C. Absolute quantitative metagenomic analysis provides more accurate insights for the anti-colitis effect of berberine via modulation of gut microbiota. Biomolecules. (2025) 15(3):400. doi: 10.3390/biom15030400, PMID: 40149936 PMC11940175

[5] Qi P Chen X Tian J Zhong K Qi Z Li M. The gut homeostasis-immune system axis: novel insights into rheumatoid arthritis pathogenesis and treatment. Front Immunol. (2024) 15:1482214. doi: 10.3389/fimmu.2024.1482214, PMID: 39391302 PMC11464316

[6] Zheng D Liwinski T Elinav E. Interaction between microbiota and immunity in health and disease. Cell Res. (2020) 30:492–506. doi: 10.1038/s41422-020-0332-7, PMID: 32433595 PMC7264227

[7] Durgan DJ. Evidence for a gut-immune-vascular axis in the development of hypertension. Acta physiologica (Oxford England). (2019) 227:e13338. doi: 10.1111/apha.13338, PMID: 31273923

[8] Mattson DL Dasinger JH Abais-Battad JM. Gut-immune-kidney axis: influence of dietary protein in salt-sensitive hypertension. Hypertension. (2022) 79:2397–408. doi: 10.1161/hypertensionaha.122.18556, PMID: 35983758 PMC9790111

[9] Dinakis E O’Donnell JA Marques FZ. The gut-immune axis during hypertension and cardiovascular diseases. Acta physiologica (Oxford England). (2024) 240:e14193. doi: 10.1111/apha.14193, PMID: 38899764

[10] Cheng H Guan X Chen D Ma W. The th17/treg cell balance: A gut microbiota-modulated story. Microorganisms. (2019) 7(12):583. doi: 10.3390/microorganisms7120583, PMID: 31756956 PMC6956175

[11] Li Y Zhang SX Yin XF Zhang MX Qiao J Xin XH. The gut microbiota and its relevance to peripheral lymphocyte subpopulations and cytokines in patients with rheumatoid arthritis. J Immunol Res. (2021) 2021:6665563. doi: 10.1155/2021/6665563, PMID: 33506059 PMC7810541

[12] Zhao T Wei Y Zhu Y Xie Z Hai Q Li Z. Gut microbiota and rheumatoid arthritis: From pathogenesis to novel therapeutic opportunities0. Front Immunol. (2022) 131007165:1007165. doi: 10.3389/fimmu.2022.1007165, PMID: 36159786 PMC9499173

[13] Wang Q Zhang SX Chang MJ Qiao J Wang CH Li XF. Characteristics of the gut microbiome and its relationship with peripheral CD4(+) T cell subpopulations and cytokines in rheumatoid arthritis. Front Microbiol. (2022) 13799602:799602. doi: 10.3389/fmicb.2022.799602, PMID: 35185845 PMC8851473

[14] Scher JU Sczesnak A Longman RS Segata N Ubeda C Bielski C. Expansion of intestinal Prevotella copri correlates with enhanced susceptibility to arthritis. Elife. (2013) 2:e01202. doi: 10.7554/eLife.01202, PMID: 24192039 PMC3816614

[15] Chen J Wright K Davis JM Jeraldo P Marietta EV Murray J. An expansion of rare lineage intestinal microbes characterizes rheumatoid arthritis. Genome Med. (2016) 8:43. doi: 10.1186/s13073-016-0299-7, PMID: 27102666 PMC4840970

[16] Maeda Y Kurakawa T Umemoto E Motooka D Ito Y Gotoh K. Dysbiosis contributes to arthritis development via activation of autoreactive T cells in the intestine. Arthritis Rheumatol (Hoboken NJ). (2016) 68:2646–61. doi: 10.1002/art.39783, PMID: 27333153

[17] Ferro M Charneca S Dourado E Guerreiro CS Fonseca JE. Probiotic supplementation for rheumatoid arthritis: A promising adjuvant therapy in the gut microbiome era. . Front Pharmacol. (2021) 12:711788. doi: 10.3389/fphar.2021.711788, PMID: 34366867 PMC8346200

[18] Honda K.Littman DR. The microbiota in adaptive immune homeostasis and disease. Nature. (2016) 535:75–84. doi: 10.1038/nature18848, PMID: 27383982

[19] Chen B Sun L Zhang X. Integration of microbiome and epigenome to decipher the pathogenesis of autoimmune diseases. J Autoimmun. (2017) 8331-:42. doi: 10.1016/j.jaut.2017.03.009, PMID: 28342734

[20] Kinashi Y.Hase K. Partners in leaky gut syndrome: intestinal dysbiosis and autoimmunity. Front Immunol. (2021) 12:673708. doi: 10.3389/fimmu.2021.673708, PMID: 33968085 PMC8100306

[21] Yu D Du J Pu X Zheng L Chen S Wang N. The gut microbiome and metabolites are altered and interrelated in patients with rheumatoid arthritis. Front Cell Infect Microbiol. (2021) 11:763507. doi: 10.3389/fcimb.2021.763507, PMID: 35145919 PMC8821809

[22] He J Chu Y Li J Meng Q Liu Y Jin J. Intestinal butyrate-metabolizing species contribute to autoantibody production and bone erosion in rheumatoid arthritis. Sci Adv. (2022) 8:eabm1511. doi: 10.1126/sciadv.abm1511, PMID: 35148177 PMC11093108

[23] Lin L Zhang K Xiong Q Zhang J Cai B Huang Z. Gut microbiota in pre-clinical rheumatoid arthritis: From pathogenesis to preventing progression. J Autoimmun. (2023) 141:103001. doi: 10.1016/j.jaut.2023.103001, PMID: 36931952

[24] Peng Y Huang Y Li H Li C Wu Y Chen ZS. Huangqin Qingre Chubi Capsule inhibits rheumatoid arthritis by regulating intestinal flora and improving intestinal barrier. Front Pharmacol. (2024) 15:1422245. doi: 10.3389/fphar.2024.1422245, PMID: 38989143 PMC11233690

[25] Pianta A Arvikar S Strle K Drouin EE Wang Q Costello CE. Evidence of the immune relevance of prevotella copri, a gut microbe, in patients with rheumatoid arthritis. Arthritis Rheumatol. (2017) 69:964–75. doi: 10.1002/art.40003, PMID: 27863183 PMC5406252

[26] Tajik N Frech M Schulz O Schälter F Lucas S Azizov V. Targeting zonulin and intestinal epithelial barrier function to prevent onset of arthritis. Nat Commun. (2020) 11:1995. doi: 10.1038/s41467-020-15831-7, PMID: 32332732 PMC7181728

[27] Flak MB Colas RA Muñoz-Atienza E Curtis MA Dalli J Pitzalis C. Inflammatory arthritis disrupts gut resolution mechanisms, promoting barrier breakdown by Porphyromonas gingivalis. JCI Insight. (2022) 7(20):e165600 . doi: 10.1172/jci.insight.165600, PMID: 36278492 PMC9744254

[28] Wu X Shou Q Chen C Cai H Zhang J Tang S. An herbal formula attenuates collagen-induced arthritis via inhibition of JAK2-STAT3 signaling and regulation of Th17 cells in mice. Oncotarget. (2017) 8:44242–54. doi: 10.18632/oncotarget.17797, PMID: 28562338 PMC5546477

[29] Li GY Tang XP Jiang Q Wang L Gong X Wang J. Five-year radiological research on bone destruction in rheumatoid arthritis treated with the formula for clearing heat and activating blood circulation. World J Integrated Traditional Western Med. (2019) 14:516–20. doi: 10.13935/j.cnki.sjzx.190417

[30] Hu Y Liu J Qi Y Zhou Q Li Y Cong C. Integrating clinical data mining, network analysis and experimental validation reveal the anti-inflammatory mechanism of Huangqin Qingre Chubi Capsule in rheumatoid arthritis treatment. J Ethnopharmacol. (2024) 329:118077. doi: 10.1016/j.jep.2024.118077, PMID: 38556141

[31] Guan YY Zhang Y Liu LX Li HD Xue D Bao WL. Suppressive effects of Wang−Bi Tab let on adjuvant−induced arthritis in rats via NF−κB and STAT3 signaling pathways. Int J Mol Med. (2018) 42:1666–74. doi: 10.3892/ijmm.2018.3723, PMID: 29901091

[32] Yang P Qian F Zhang M Xu AL Wang X Jiang B. Zishen Tongluo formula ameliorates collagen-induced arthritis in mice by modulation of Th17/Treg balance. J Ethnopharmacol. (2020) 250:112428. doi: 10.1016/j.jep.2019.112428, PMID: 31783137

[33] He YF Mai CT Pan HD Liu L Zhou H Xie Y. Targeting immunometabolism by active ingredients derived from traditional Chinese medicines for treatment of rheumatoid arthritis. Chin Herb Med. (2021) 13:451–60. doi: 10.1016/j.chmed.2021.09.005, PMID: 36119361 PMC9476673

[34] Wang T Lin J Di SN Kuang HX. Clinical efficacy observation of Wutou Decoction and its disassembled prescriptions on TNF-α and IL-6 in 60 cases of rheumatoid arthritis with cold-damp syndrome. Acta Chin Med Pharmacol. (2016) 44:85–7. doi: 10.19664/j.cnki.1002-2392.2016.01.027

[35] Daily JW Zhang T Cao S Park S. Efficacy and safety of guiZhi-shaoYao-zhiMu decoction for treating rheumatoid arthritis: A systematic review and meta-analysis of randomized clinical trials. J Altern complementary Med (New York NY). (2017) 23:756–70. doi: 10.1089/acm.2017.0098, PMID: 28609129

[36] Yang M Guo MY Luo Y Yun MD Yan J Liu T. Effect of Artemisia annua extract on treating active rheumatoid arthritis: A randomized controlled trial. Chin J Integr Med. (2017) 23:496–503. doi: 10.1007/s11655-016-2650-7, PMID: 28035541

[37] Zhou YY Xia X Peng WK Wang QH Peng JH Li YL. The effectiveness and safety of tripterygium wilfordii hook. F extracts in rheumatoid arthritis: A systematic review and meta-analysis. Front Pharmacol. (2018) 9:356. doi: 10.3389/fphar.2018.00356, PMID: 29713281 PMC5911475

[38] Aryaeian N Hadidi M Mahmoudi M Asgari M Hezaveh ZS Sadehi SK. The effect of black barberry hydroalcoholic extract on immune mediators in patients with active rheumatoid arthritis: A randomized, double-blind, controlled clinical trial. Phytother Res. (2021) 35:1062–8. doi: 10.1002/ptr.6874, PMID: 32914483

[39] Li S Liu D Chen Z Wei S Xu W Li X. Comparative efficacy and safety of four classical prescriptions for clearing damp-heat recommended by clinical guidelines in treating rheumatoid arthritis: a network meta-analysis. Ann palliative Med. (2021) 10:7298–328. doi: 10.21037/apm-21-445, PMID: 34353032

[40] Wang FZ Li J Song WH Guan QCR. Clinical curative observation of using Chinese herb Danggui Niantong decoction combined with Western medicine in the treatment of early stage rheumatoid arthritis of dampness–heat blockage syndrome. J Sichuan Traditional Chin Med. (2018) 36:145–8.

[41] Zhai XJ. Exploring the therapeutic effect of methotrexate combined with tripterygium wilfordii polyglycoside tab lets in the treatment of rheumatoid arthritis. China Pract Med. (2019) 14:107–8. doi: 10.14163/j.cnki.11-5547/r.2019.21.061

[42] Zhao Y Hu J Zhang GM Guan YX Cheng GL Qu HH. Origin and application of Jingfang Baidu powder. Global Traditional Chin Med. (2020) 13:1996–2002.

[43] Wang Y Chen S Du K Liang C Wang S Owusu Boadi E. Traditional herbal medicine: Therapeutic potential in rheumatoid arthritis. J Ethnopharmacol. (2021) 279:114368. doi: 10.1016/j.jep.2021.114368, PMID: 34197960

[44] Zhang R Tang XP Wang J Liu WX Liu J Wang Y. The impact of traditional chinese medicine qingreHuoxue treatment and the combination of methotrexate and hydroxychloroquine on the radiological progression of active rheumatoid arthritis: A 52-week follow-up of a randomized controlled clinical study. Evid Based Complement Alternat Med. (2022) 2022:5808400. doi: 10.1155/2022/5808400, PMID: 35463097 PMC9019417

[45] Ge L Shi ZM. Randomized controlled study of Danggui Niantong decoction combined with methotrexate in the treatment of rheumatoid arthritis. Acta Chin Med Pharmacol. (2017) 45:84–6. doi: 10.19664/j.cnki.1002-2392.2017.02.025

[46] Luo J Jin DE Yang GY Zhang YZ Wang JM Kong WP. Total glucosides of paeony for rheumatoid arthritis: A systematic review of randomized controlled trials. Complementary therapies Med. (2017) 3446-:56. doi: 10.1016/j.ctim.2017.07.010, PMID: 28917375

[47] Aryaeian N Shahram F Mahmoudi M Tavakoli H Yousefi B Arablou T. The effect of ginger supplementation on some immunity and inflammation intermediate genes expression in patients with active Rheumatoid Arthritis. Gene. (2019) 698179-:185. doi: 10.1016/j.gene.2019.01.048, PMID: 30844477

[48] Li Y Goronzy JJ Weyand CM. DNA damage, metabolism and aging in pro-inflammatory T cells: Rheumatoid arthritis as a model system. Exp gerontology. (2018) 105118-:127. doi: 10.1016/j.exger.2017.10.027, PMID: 29101015 PMC5871568

[49] Jang S Kwon EJ Lee JJ. Rheumatoid arthritis: pathogenic roles of diverse immune cells. Int J Mol Sci. (2022) 23(2):905. doi: 10.3390/ijms23020905, PMID: 35055087 PMC8780115

[50] Vitales-Noyola M Hernández-Castro B Alvarado-Hernández D Baranda L Bernal-Silva S Abud-Mendoza C. Levels of pathogenic th17 and th22 cells in patients with rheumatoid arthritis. J Immunol Res. (2022) 2022:5398743. doi: 10.1155/2022/5398743, PMID: 35996623 PMC9392632

[51] Bao Y Peng J Yang KL Wang CH Guo YF Guo ZS. Therapeutic effects of Chinese medicine Di-Long (Pheretima vulgaris) on rheumatoid arthritis through inhibiting NF-κB activation and regulating Th1/Th2 balance. BioMed Pharmacother. (2022) 147:112643. doi: 10.1016/j.biopha.2022.112643, PMID: 35033948

[52] Kuwabara T Ishikawa F Kondo M Kakiuchi T. The role of IL-17 and related cytokines in inflammatory autoimmune diseases. Mediators Inflammation. (2017) 2017:3908061. doi: 10.1155/2017/3908061, PMID: 28316374 PMC5337858

[53] Li W Yu L Li W Ge G Ma Y Xiao L. Prevention and treatment of inflammatory arthritis with traditional Chinese medicine: Underlying mechanisms based on cell and molecular targets. Ageing Res Rev. (2023) 89:101981. doi: 10.1016/j.arr.2023.101981, PMID: 37302756

[54] Xu H Zhao H Lu C Qiu Q Wang G Huang J. Triptolide inhibits osteoclast differentiation and bone resorption *in vitro* via enhancing the production of IL-10 and TGF-β1 by regulatory T cells. Mediators Inflammation. (2016) 2016:8048170. doi: 10.1155/2016/8048170, PMID: 27413257 PMC4930824

[55] Cañete JD Martínez SE Farrés J Sanmartí R Blay M Gómez A. Differential Th1/Th2 cytokine patterns in chronic arthritis: interferon gamma is highly expressed in synovium of rheumatoid arthritis compared with seronegative spondyloarthropathies. Ann rheumatic Dis. (2000) 59:263–8. doi: 10.1136/ard.59.4.263, PMID: 10733472 PMC1753106

[56] Mariani FM Martelli I Pistone F Chericoni E Puxeddu I Alunno A. Pathogenesis of rheumatoid arthritis: one year in review 2023. Clin Exp Rheumatol. (2023) 41:1725–34. doi: 10.55563/clinexprheumatol/sgjk6e, PMID: 37497721

[57] Rao DA Gurish MF Marshall JL Slowikowski K Fonseka CY Liu Y. Pathologically expanded peripheral T helper cell subset drives B cells in rheumatoid arthritis. Nature. (2017) 542:110–4. doi: 10.1038/nature20810, PMID: 28150777 PMC5349321

[58] Yoshitomi H.Ueno H. Shared and distinct roles of T peripheral helper and T follicular helper cells in human diseases. Cell Mol Immunol. (2021) 18:523–7. doi: 10.1038/s41423-020-00529-z, PMID: 32868910 PMC8027819

[59] Ardura JA Rackov G Izquierdo E Alonso V Gortazar AR Escribese MM. Targeting macrophages: friends or foes in disease? Front Pharmacol. (2019) 10:1255. doi: 10.3389/fphar.2019.01255, PMID: 31708781 PMC6819424

[60] Tu J Hong W Zhang P Wang X Körner H Wei W. Ontology and function of fibroblast-like and macrophage-like synoviocytes: how do they talk to each other and can they be targeted for rheumatoid arthritis therapy? Front Immunol. (2018) 9:1467. doi: 10.3389/fimmu.2018.01467, PMID: 29997624 PMC6028561

[61] Nygaard G.Firestein GS. Restoring synovial homeostasis in rheumatoid arthritis by targeting fibroblast-like synoviocytes. Nat Rev Rheumatol. (2020) 16:316–33. doi: 10.1038/s41584-020-0413-5, PMID: 32393826 PMC7987137

[62] Khandpur R Carmona-Rivera C Vivekanandan-Giri A Gizinski A Yalavarthi S Knight JS. NETs are a source of citrullinated autoantigens and stimulate inflammatory responses in rheumatoid arthritis. Sci Transl Med. (2013) 5:178ra40. htt. doi: 10.1126/scitranslmed.3005580, PMID: 23536012 PMC3727661

[63] O’Neil LJ Oliveira CB Wang X Navarrete M Barrera-Vargas A Merayo-Chalico J. Neutrophil extracellular trap-associated carbamylation and histones trigger osteoclast formation in rheumatoid arthritis. Ann rheumatic Dis. (2023) 82:630–8. doi: 10.1136/ard-2022-223568, PMID: 36737106 PMC11302494

[64] Clavel C Nogueira L Laurent L Iobagiu C Vincent C Sebbag M. Induction of macrophage secretion of tumor necrosis factor alpha through Fcgamma receptor IIa engagement by rheumatoid arthritis-specific autoantibodies to citrullinated proteins complexed with fibrinogen. Arthritis rheumatism. (2008) 58:678–88. doi: 10.1002/art.23284, PMID: 18311806

[65] Sokolove J Zhao X Chandra PE Robinson WH. Immune complexes containing citrullinated fibrinogen costimulate macrophages via Toll-like receptor 4 and Fcγ receptor. Arthritis rheumatism. (2011) 63:53–62. doi: 10.1002/art.30081, PMID: 20954191 PMC3015008

[66] Zhao T Xie Z Xi Y Liu L Li Z Qin D. How to model rheumatoid arthritis in animals: from rodents to non-human primates. Front Immunol. (2022) 13:887460. doi: 10.3389/fimmu.2022.887460, PMID: 35693791 PMC9174425

[67] Månsson I Norberg R Olhagen B Björklund NE. Arthritis in pigs induced by dietary factors. Microbiologic, clinical and histologic studies. Clin Exp Immunol. (1971) 9:677–93., PMID: 4335983 PMC1713057

[68] Liu X Zeng B Zhang J Li W Mou F Wang H. Role of the gut microbiome in modulating arthritis progression in mice. Sci Rep. (2016) 6:30594. doi: 10.1038/srep30594, PMID: 27481047 PMC4969881

[69] Huang Y Li M Zhou L Xu D Qian F Zhang J. Effects of qingluo tongbi decoction on gut flora of rats with adjuvant-induced arthritis and the underlying mechanism. Evid Based Complement Alternat Med. (2019) 2019:6308021. doi: 10.1155/2019/6308021, PMID: 31531116 PMC6721445

[70] Yue M Tao Y Fang Y Lian X Zhang Q Xia Y. The gut microbiota modulator berberine ameliorates collagen-induced arthritis in rats by facilitating the generation of butyrate and adjusting the intestinal hypoxia and nitrate supply. FASEB journal: Off Publ Fed Am Societies Exp Biol. (2019) 33:12311–23. doi: 10.1096/fj.201900425RR, PMID: 31425655 PMC6902671

[71] Kishikawa T Maeda Y Nii T Motooka D Matsumoto Y Matsushita M. Metagenome-wide association study of gut microbiome revealed novel aetiology of rheumatoid arthritis in the Japanese population. Ann rheumatic Dis. (2020) 79:103–11. doi: 10.1136/annrheumdis-2019-215743, PMID: 31699813 PMC6937407

[72] Xu X Wang M Wang Z Chen Q Chen X Xu Y. The bridge of the gut-joint axis: Gut microbial metabolites in rheumatoid arthritis. Front Immunol. (2022) 131007610:1007610. doi: 10.3389/fimmu.2022.1007610, PMID: 36275747 PMC9583880

[73] Rosser EC Piper CJM Matei DE Blair PA Rendeiro AF Orford M. Microbiota-derived metabolites suppress arthritis by amplifying aryl-hydrocarbon receptor activation in regulatory B cells. Cell Metab. (2020) 31:837–851.e10. doi: 10.1016/j.cmet.2020.03.003, PMID: 32213346 PMC7156916

[74] Li C Liang Y Qiao Y. Messengers from the gut: gut microbiota-derived metabolites on host regulation. Front Microbiol. (2022) 13:863407. doi: 10.3389/fmicb.2022.863407, PMID: 35531300 PMC9073088

[75] Lu ZF Hsu CY Younis NK Mustafa MA Matveeva EA Al-Juboory YHO. Exploring the significance of microbiota metabolites in rheumatoid arthritis: uncovering their contribution from disease development to biomarker potential. APMIS: Acta pathologica microbiologica immunologica Scandinavica. (2024) 132:382–415. doi: 10.1111/apm.13401, PMID: 38469726

[76] Gumà UM. Modelos animales en la artritis reumatoide. Reumatología clínica. (2008) 4:129–31. doi: 10.1016/S1699-258X(08)71820-5, PMID: 21794517

[77] Aa LX Fei F Qi Q Sun RB Gu SH Di ZZ. Rebalancing of the gut flora and microbial metabolism is responsible for the anti-arthritis effect of kaempferol. Acta pharmacologica Sin. (2020) 41:73–81. doi: 10.1038/s41401-019-0279-8, PMID: 31427695 PMC7468310

[78] Abdollahi-Roodsaz S Joosten LA Koenders MI Devesa I Roelofs MF Radstake TR. Stimulation of TLR2 and TLR4 differentially skews the balance of T cells in a mouse model of arthritis. J Clin Invest. (2008) 118:205–16. doi: 10.1172/jci32639, PMID: 18060042 PMC2104479

[79] Liu X Zou Q Zeng B Fang Y Wei H. Analysis of fecal Lactobacillus community structure in patients with early rheumatoid arthritis. Curr Microbiol. (2013) 67:170–6. doi: 10.1007/s00284-013-0338-1, PMID: 23483307

[80] Marazzato M Iannuccelli C Guzzo MP Nencioni L Lucchino B Radocchia G. Gut microbiota structure and metabolites, before and after treatment in early rheumatoid arthritis patients: A pilot study. Front Med. (2022) 9:921675. doi: 10.3389/fmed.2022.921675, PMID: 35872763 PMC9304627

[81] Meng Q Lin M Song W Wu J Cao G Huang P. The gut-joint axis mediates the TNF-induced RA process and PBMT therapeutic effects through the metabolites of gut microbiota. Gut Microbes. (2023) 15:2281382. doi: 10.1080/19490976.2023.2281382, PMID: 38017660 PMC10730145

[82] Zhang X Zhang D Jia H Feng Q Wang D Liang D. The oral and gut microbiomes are perturbed in rheumatoid arthritis and partly normalized after treatment. Nat Med. (2015) 21:895–905. doi: 10.1038/nm.3914, PMID: 26214836

[83] Chen Y Ma C Liu L He J Zhu C Zheng F. Analysis of gut microbiota and metabolites in patients with rheumatoid arthritis and identification of potential biomarkers. Aging. (2021) 13:23689–701. doi: 10.18632/aging.203641, PMID: 34670873 PMC8580343

[84] Konig MF Abusleme L Reinholdt J Palmer RJ Teles RP Sampson K. Aggregatibacter actinomycetemcomitans-induced hypercitrullination links periodontal infection to autoimmunity in rheumatoid arthritis. Sci Transl Med. (2016) 8(369):369ra176. doi: 10.1126/scitranslmed.aaj1921, PMID: 27974664 PMC5384717

[85] Blenkinsopp HC Seidler K Barrow M. Microbial imbalance and intestinal permeability in the pathogenesis of rheumatoid arthritis: A mechanism review with a focus on bacterial translocation, citrullination, and probiotic intervention. J Am Nutr Assoc. (2024) 43:59–76. doi: 10.1080/27697061.2023.2211129, PMID: 37294082

[86] Malmström V Catrina AI Klareskog L. The immunopathogenesis of seropositive rheumatoid arthritis: from triggering to targeting. Nat Rev Immunol. (2017) 17:60–75. doi: 10.1038/nri.2016.124, PMID: 27916980

[87] Bennike TB Ellingsen T Glerup H Bonderup OK Carlsen TG Meyer MK. Proteome analysis of rheumatoid arthritis gut mucosa. J Proteome Res. (2017) 16:346–54. doi: 10.1021/acs.jproteome.6b00598, PMID: 27627584

[88] Lee N.Kim WU. Microbiota in T-cell homeostasis and inflammatory diseases. Exp Mol Med. (2017) 49:e340. doi: 10.1038/emm.2017.36, PMID: 28546563 PMC5454441

[89] Su R Li B Wu R Xie Y Gao A Gao C. Stratified distribution of Th17 and Treg cells in patients with multi-stage rheumatoid arthritis. Arthritis Res Ther. (2023) 25:55. doi: 10.1186/s13075-023-03041-7, PMID: 37016395 PMC10071616

[90] de Vos P Mujagic Z de Haan BJ Siezen RJ Bron PA Meijerink M. Lactobacillus plantarum strains can enhance human mucosal and systemic immunity and prevent non-steroidal anti-inflammatory drug induced reduction in T regulatory cells. Front Immunol. (2017) 81000:1000. doi: 10.3389/fimmu.2017.01000, PMID: 28878772 PMC5572349

[91] Hosoya T Sakai F Yamashita M Shiozaki T Endo T Ukibe K. Lactobacillus helveticus SBT2171 inhibits lymphocyte proliferation by regulation of the JNK signaling pathway. PloS One. (2014) 9:e108360. doi: 10.1371/journal.pone.0108360, PMID: 25268890 PMC4182466

[92] Atarashi K Tanoue T Shima T Imaoka A Kuwahara T Momose Y. Induction of colonic regulatory T cells by indigenous Clostridium species. Science. (2011) 331:337–41. doi: 10.1126/science.1198469, PMID: 21205640 PMC3969237

[93] Round JL Lee SM Li J Tran G Jabri B Chatila TA. The Toll-like receptor 2 pathway establishes colonization by a commensal of the human microbiota. Science. (2011) 332:974–7. doi: 10.1126/science.1206095, PMID: 21512004 PMC3164325

[94] Telesford KM Yan W Ochoa-Reparaz J Pant A Kircher C Christy MA. A commensal symbiotic factor derived from Bacteroides fragilis promotes human CD39(+)Foxp3(+) T cells and Treg function. Gut Microbes. (2015) 6:234–42. doi: 10.1080/19490976.2015.1056973, PMID: 26230152 PMC4615798

[95] Bai Y Li Y Marion T Tong Y Zaiss MM Tang Z. Resistant starch intake alleviates collagen-induced arthritis in mice by modulating gut microbiota and promoting concomitant propionate production. J Autoimmun. (2021) 116:102564. doi: 10.1016/j.jaut.2020.102564, PMID: 33203617

[96] Wu HJ Ivanov II Darce J Hattori K Shima T Umesaki Y. Gut-residing segmented filamentous bacteria drive autoimmune arthritis via T helper 17 cells. Immunity. (2010) 32:815–27. doi: 10.1016/j.immuni.2010.06.001, PMID: 20620945 PMC2904693

[97] Atarashi K Tanoue T Ando M Kamada N Nagano Y Narushima S. Th17 cell induction by adhesion of microbes to intestinal epithelial cells. Cell. (2015) 163:367–80. doi: 10.1016/j.cell.2015.08.058, PMID: 26411289 PMC4765954

[98] Ravindran R Loebbermann J Nakaya HI Khan N Ma H Gama L. The amino acid sensor GCN2 controls gut inflammation by inhibiting inflammasome activation. Nature. (2016) 531:523–7. doi: 10.1038/nature17186, PMID: 26982722 PMC4854628

[99] Teng F Klinger CN Felix KM Bradley CP Wu E Tran NL. Gut microbiota drive autoimmune arthritis by promoting differentiation and migration of peyer’s patch T follicular helper cells. Immunity. (2016) 44:875–88. doi: 10.1016/j.immuni.2016.03.013, PMID: 27096318 PMC5296410

[100] Block KE Zheng Z Dent AL Kee BL Huang H. Gut Microbiota Regulates K/BxN Autoimmune Arthritis through Follicular Helper T but Not Th17 Cells. J Immunol (Baltimore Md: 1950). (2016) 196:1550–7. doi: 10.4049/jimmunol.1501904, PMID: 26783341 PMC4744513

[101] Monach PA Mathis D Benoist C. The K/BxN arthritis model. Curr Protoc Immunol Chapter. (2008) 1515.22.1-:15.22.12. doi: 10.1002/0471142735.im1522s81, PMID: 18491295

[102] Blagojević V Kovačević-Jovanović V Ćuruvija I Petrović R Vujnović I Vujić V. Rat strain differences in peritoneal immune cell response to selected gut microbiota: A crossroad between tolerance and autoimmunity? Life Sci. (2018) 197147-:157. doi: 10.1016/j.lfs.2018.02.011, PMID: 29427649

[103] Tanoue T Morita S Plichta DR Skelly AN Suda W Sugiura Y. A defined commensal consortium elicits CD8 T cells and anti-cancer immunity. Nature. (2019) 565:600–5. doi: 10.1038/s41586-019-0878-z, PMID: 30675064

[104] Zhou L Chu C Teng F Bessman NJ Goc J Santosa EK. Innate lymphoid cells support regulatory T cells in the intestine through interleukin-2. Nature. (2019) 568:405–9. doi: 10.1038/s41586-019-1082-x, PMID: 30944470 PMC6481643

[105] Ye H Wang H Han B Chen K Wang X Ma F. Guizhi Shaoyao Zhimu decoction inhibits neutrophil extracellular traps formation to relieve rheumatoid arthritis via gut microbial outer membrane vesicles. Phytomedicine. (2025) 136:156254. doi: 10.1016/j.phymed.2024.156254, PMID: 39586125

[106] Qiao J Zhang SX Chang MJ Cheng T Zhang JQ Zhao R. Specific enterotype of gut microbiota predicted clinical effect of methotrexate in patients with rheumatoid arthritis. Rheumatology. (2023) 62:1087–96. doi: 10.1093/rheumatology/keac458, PMID: 35946529

[107] Wang X Liu D Li D Yan J Yang J Zhong X. Combined treatment with glucosamine and chondroitin sulfate improves rheumatoid arthritis in rats by regulating the gut microbiota. Nutr Metab. (2023) 20:22. doi: 10.1186/s12986-023-00735-2, PMID: 37016458 PMC10071728

[108] Amdekar S Singh V Kumar A Sharma P Singh R. Lactobacillus casei and Lactobacillus acidophilus regulate inflammatory pathway and improve antioxidant status in collagen-induced arthritic rats. J Interferon Cytokine research: Off J Int Soc Interferon Cytokine Res. (2013) 33:1–8. doi: 10.1089/jir.2012.0034, PMID: 23030670

[109] Sokol H Pigneur B Watterlot L Lakhdari O Bermúdez-Humarán LG Gratadoux JJ. Faecalibacterium prausnitzii is an anti-inflammatory commensal bacterium identified by gut microbiota analysis of Crohn disease patients. Proc Natl Acad Sci U.S.A. (2008) 105:16731–6. doi: 10.1073/pnas.0804812105, PMID: 18936492 PMC2575488

[110] Kawano M Miyoshi M Miyazaki T. Lactobacillus helveticus SBT2171 Induces A20 Expression via Toll-Like Receptor 2 Signaling and Inhibits the Lipopolysaccharide-Induced Activation of Nuclear Factor-kappa B and Mitogen-Activated Protein Kinases in Peritoneal Macrophages. . Front Immunol. (2019) 10:845. doi: 10.3389/fimmu.2019.00845, PMID: 31057558 PMC6478895

[111] Jiang L Shang M Yu S Liu Y Zhang H Zhou Y. A high-fiber diet synergizes with Prevotella copri and exacerbates rheumatoid arthritis. Cell Mol Immunol. (2022) 19:1414–24. doi: 10.1038/s41423-022-00934-6, PMID: 36323929 PMC9709035

[112] Yao Y Cai X Zheng Y Zhang M Fei W Sun D. Short-chain fatty acids regulate B cells differentiation via the FFA2 receptor to alleviate rheumatoid arthritis. Br J Pharmacol. (2022) 179:4315–29. doi: 10.1111/bph.15852, PMID: 35393660

[113] Piper CJM Rosser EC Oleinika K Nistala K Krausgruber T Rendeiro AF. Aryl hydrocarbon receptor contributes to the transcriptional program of IL-10-producing regulatory B cells. Cell Rep. (2019) 29:1878–1892.e7. doi: 10.1016/j.celrep.2019.10.018, PMID: 31722204 PMC6856759

[114] Lee S Koh J Chang Y Kim HY Chung DH. Invariant NKT cells functionally link microbiota-induced butyrate production and joint inflammation. J Immunol (Baltimore Md. (2019) 1950) 203:3199–208. doi: 10.4049/jimmunol.1801314, PMID: 31732526

[115] Yang W.Cong Y. Gut microbiota-derived metabolites in the regulation of host immune responses and immune-related inflammatory diseases. Cell Mol Immunol. (2021) 18:866–77. doi: 10.1038/s41423-021-00661-4, PMID: 33707689 PMC8115644

[116] Feng W Wu Y Chen G Fu S Li B Huang B. Sodium butyrate attenuates diarrhea in weaned piglets and promotes tight junction protein expression in colon in a GPR109A-dependent manner. Cell Physiol Biochem. (2018) 47:1617–29. doi: 10.1159/000490981, PMID: 29949795

[117] Lv J Hao P Zhou Y Liu T Wang L Song C. Role of the intestinal flora-immunity axis in the pathogenesis of rheumatoid arthritis—mechanisms regulating short-chain fatty acids and Th17/Treg homeostasis. Mol Biol Rep. (2025) 52:617. doi: 10.1007/s11033-025-10714-w, PMID: 40544212

[118] Zhang D.Frenette PS. Cross talk between neutrophils and the microbiota. Blood. (2019) 133:2168–77. doi: 10.1182/blood-2018-11-844555, PMID: 30898860 PMC6524562

[119] Man AWC Zhou Y Xia N Li H. Involvement of gut microbiota, microbial metabolites and interaction with polyphenol in host immunometabolism. Nutrients. (2020) 12(10):3054. doi: 10.3390/nu12103054, PMID: 33036205 PMC7601750

[120] Wang Y Wei J Zhang W Doherty M Zhang Y Xie H. Gut dysbiosis in rheumatic diseases: A systematic review and meta-analysis of 92 observational studies. EBioMedicine. (2022) 80:104055. doi: 10.1016/j.ebiom.2022.104055, PMID: 35594658 PMC9120231

[121] Hills RD Jr. Pontefract BA Mishcon HR Black CA Sutton SC Theberge CR. Gut microbiome: profound implications for diet and disease. Nutrients. (2019) 11(7):1613. doi: 10.3390/nu11071613, PMID: 31315227 PMC6682904

[122] Jiao Y Wu L Huntington ND Zhang X. Crosstalk between gut microbiota and innate immunity and its implication in autoimmune diseases. Front Immunol. (2020) 11282:282. doi: 10.3389/fimmu.2020.00282, PMID: 32153586 PMC7047319

[123] Smith PM Howitt MR Panikov N Michaud M Gallini CA Bohlooly YM. The microbial metabolites, short-chain fatty acids, regulate colonic Treg cell homeostasis. Science. (2013) 341:569–73. doi: 10.1126/science.1241165, PMID: 23828891 PMC3807819

[124] Fan Z Yang B Ross RP Stanton C Shi G Zhao J. Protective effects of Bifidobacterium adolescentis on collagen-induced arthritis in rats depend on timing of administration. Food Funct. (2020) 11:4499–511. doi: 10.1039/d0fo00077a, PMID: 32383727

[125] Yang W Yu T Cong Y. CD4(+) T cell metabolism, gut microbiota, and autoimmune diseases: implication in precision medicine of autoimmune diseases. Precis Clin Med. (2022) 5:pbac018. doi: 10.1093/pcmedi/pbac018, PMID: 35990897 PMC9384833

[126] Bian Z Zhang Q Qin Y Sun X Liu L Liu H. Sodium butyrate inhibits oxidative stress and NF-κB/NLRP3 activation in dextran sulfate sodium salt-induced colitis in mice with involvement of the Nrf2 signaling pathway and mitophagy. Digestive Dis Sci. (2023) 68:2981–96. doi: 10.1007/s10620-023-07845-0, PMID: 36867295

[127] Liao H Zheng J Lu J Shen HL. NF-κB signaling pathway in rheumatoid arthritis: mechanisms and therapeutic potential. Mol Neurobiol. (2025) 62:6998–7021. doi: 10.1007/s12035-024-04634-2, PMID: 39560902

[128] Liu T Sun Z Yang Z Qiao X. Microbiota-derived short-chain fatty acids and modulation of host-derived peptides formation: focused on host defense peptides. Biomedicine pharmacotherapy. (2023) 162:114586. doi: 10.1016/j.biopha.2023.114586, PMID: 36989711

[129] Sun H Guo Y Wang H Yin A Hu J Yuan T. Gut commensal Parabacteroides distasonis alleviates inflammatory arthritis. Gut. (2023) 72:1664–77. doi: 10.1136/gutjnl-2022-327756, PMID: 36604114

[130] Murayama MA Kakuta S Inoue A Umeda N Yonezawa T Maruhashi T. CTRP6 is an endogenous complement regulator that can effectively treat induced arthritis. Nat Commun. (2015) 6:8483. doi: 10.1038/ncomms9483, PMID: 26404464 PMC4598845

[131] Mazmanian SK Liu CH Tzianabos AO Kasper DL. An immunomodulatory molecule of symbiotic bacteria directs maturation of the host immune system. Cell. (2005) 122:107–18. doi: 10.1016/j.cell.2005.05.007, PMID: 16009137

[132] Round J.L.Mazmanian SK. Inducible Foxp3+ regulatory T-cell development by a commensal bacterium of the intestinal microbiota. Proc Natl Acad Sci U.S.A. (2010) 107:12204–9. doi: 10.1073/pnas.0909122107, PMID: 20566854 PMC2901479

[133] Van Itallie CM Fanning AS Bridges A Anderson JM. ZO-1 stabilizes the tight junction solute barrier through coupling to the perijunctional cytoskeleton. Mol Biol Cell. (2009) 20:3930–40. doi: 10.1091/mbc.e09-04-0320, PMID: 19605556 PMC2735491

[134] Chiang HI Li JR Liu CC Liu PY Chen HH Chen YM. An association of gut microbiota with different phenotypes in chinese patients with rheumatoid arthritis. J Clin Med. (2019) 8(11):1770. doi: 10.3390/jcm8111770, PMID: 31652955 PMC6912313

[135] Parantainen J Barreto G Koivuniemi R Kautiainen H Nordström D Moilanen E. The biological activity of serum bacterial lipopolysaccharides associates with disease activity and likelihood of achieving remission in patients with rheumatoid arthritis. Arthritis Res Ther. (2022) 24:256. doi: 10.1186/s13075-022-02946-z, PMID: 36411473 PMC9677706

[136] Brandl C Bucci L Schett G Zaiss MM. Crossing the barriers: Revisiting the gut feeling in rheumatoid arthritis. Eur J Immunol. (2021) 51:798–810. doi: 10.1002/eji.202048876, PMID: 33594693

[137] Zhou L Zhang M Wang Y Dorfman RG Liu H Yu T. Faecalibacterium prausnitzii produces butyrate to maintain th17/treg balance and to ameliorate colorectal colitis by inhibiting histone deacetylase 1. Inflammatory bowel Dis. (2018) 24:1926–40. doi: 10.1093/ibd/izy182, PMID: 29796620

[138] Plovier H Everard A Druart C Depommier C Van Hul M Geurts L. A purified membrane protein from Akkermansia muciniphila or the pasteurized bacterium improves metabolism in obese and diabetic mice. Nat Med. (2017) 23:107–13. doi: 10.1038/nm.4236, PMID: 27892954

[139] Desai MS Seekatz AM Koropatkin NM Kamada N Hickey CA Wolter M. A dietary fiber-deprived gut microbiota degrades the colonic mucus barrier and enhances pathogen susceptibility. Cell. (2016) 167:1339–1353.e21. doi: 10.1016/j.cell.2016.10.043, PMID: 27863247 PMC5131798

[140] Khan S Waliullah S Godfrey V Khan MAW Ramachandran RA Cantarel BL. Dietary simple sugars alter microbial ecology in the gut and promote colitis in mice. Sci Transl Med. (2020) 12(5):1339–53.e21. doi: 10.1126/scitranslmed.aay6218, PMID: 33115951

[141] Matei DE Menon M Alber DG Smith AM Nedjat-Shokouhi B Fasano A. Intestinal barrier dysfunction plays an integral role in arthritis pathology and can be targeted to ameliorate disease. Med (New York NY). (2021) 2:864–883.e9. doi: 10.1016/j.medj.2021.04.013, PMID: 34296202 PMC8280953

[142] Fachi JL Felipe JS Pral LP da Silva BK Corrêa RO de Andrade MCP. Butyrate Protects Mice from Clostridium difficile-Induced Colitis through an HIF-1-Dependent Mechanism. Cell Rep. (2019) 27:750–761.e7. doi: 10.1016/j.celrep.2019.03.054, PMID: 30995474

[143] Venkatesh M Mukherjee S Wang H Li H Sun K Benechet AP. Symbiotic bacterial metabolites regulate gastrointestinal barrier function via the xenobiotic sensor PXR and Toll-like receptor 4. Immunity. (2014) 41:296–310. doi: 10.1016/j.immuni.2014.06.014, PMID: 25065623 PMC4142105

[144] Singh R Chandrashekharappa S Bodduluri SR Baby BV Hegde B Kotla NG. Enhancement of the gut barrier integrity by a microbial metabolite through the Nrf2 pathway. Nat Commun. (2019) 10:89. doi: 10.1038/s41467-018-07859-7, PMID: 30626868 PMC6327034

[145] Kishino S Takeuchi M Park SB Hirata A Kitamura N Kunisawa J. Polyunsaturated fatty acid saturation by gut lactic acid bacteria affecting host lipid composition. Proc Natl Acad Sci U.S.A. (2013) 110:17808–13. doi: 10.1073/pnas.1312937110, PMID: 24127592 PMC3816446

[146] Miyamoto J Mizukure T Park SB Kishino S Kimura I Hirano K. A gut microbial metabolite of linoleic acid, 10-hydroxy-cis-12-octadecenoic acid, ameliorates intestinal epithelial barrier impairment partially via GPR40-MEK-ERK pathway. J Biol Chem. (2015) 290:2902–18. doi: 10.1074/jbc.M114.610733, PMID: 25505251 PMC4317025

[147] Negi S Singh H Mukhopadhyay A. Gut bacterial peptides with autoimmunity potential as environmental trigger for late onset complex diseases: In-silico study. PloS One. (2017) 12:e0180518. doi: 10.1371/journal.pone.0180518, PMID: 28678867 PMC5498033

[148] Pianta A Arvikar SL Strle K Drouin EE Wang Q Costello CE. Two rheumatoid arthritis-specific autoantigens correlate microbial immunity with autoimmune responses in joints. J Clin Invest. (2017) 127:2946–56. doi: 10.1172/jci93450, PMID: 28650341 PMC5531397

[149] Zhang Y Bai M Zhang B Liu C Guo Q Sun Y. Uncovering pharmacological mechanisms of Wu-tou decoction acting on rheumatoid arthritis through systems approaches: drug-target prediction, network analysis and experimental validation. Sci Rep. (2015) 5:9463. doi: 10.1038/srep09463, PMID: 25820382 PMC4377576

[150] Wang T Lin J Di SN Kuang HX. Clinical efficacy observation of wutou decoction and its disassembled prescriptions on 116 cases of rheumatoid arthritis with cold-dampness type. Lishizhen Med Materia Med Res. (2016) 27 145–146.

[151] Cheng X Pi Z Zheng Z Liu S Song F Liu Z. Combined 16S rRNA gene sequencing and metabolomics to investigate the protective effects of Wu-tou decoction on rheumatoid arthritis in rats. J Chromatogr B Analytical Technol Biomed Life Sci. (2022) 1199:123249. doi: 10.1016/j.jchromb.2022.123249, PMID: 35447521

[152] Lin W Shen P Huang Y Han L Ba X Huang Y. Wutou decoction attenuates the synovial inflammation of collagen-induced arthritis rats via regulating macrophage M1/M2 type polarization. J Ethnopharmacol. (2023) 301:115802. doi: 10.1016/j.jep.2022.115802, PMID: 36209953

[153] Wang PJ Shi XG Ge Z Peng XF Huang ZS. Effects of wutou decoction and its compatibility on peripheral blood T lymphocytes in rats with adjuvant arthritis. Pharmacol Clinics Chin Materia Med. (2007) 03):9–10.

[154] Xie Y Mai CT Zheng DC He YF Feng SL Li YZ. Wutou decoction ameliorates experimental rheumatoid arthritis via regulating NF-kB and Nrf2: Integrating efficacy-oriented compatibility of traditional Chinese medicine. Phytomedicine. (2021) 85:153522. doi: 10.1016/j.phymed.2021.153522, PMID: 33799223

[155] Wu ZY Cao W Qi X Wang HX Zhi K Wu GJ. Effect of qingre huoxue recipe on intestinal microecology and th17/treg balance in collagen induced arthritis rats. Chin J Integrated Traditional Western Med. (2018) 38:681–6.

[156] Yao LS Fu ZX Fan FY. Clinical Observation of Danggui Niantong Tang in the Treatment of Rheumatoid Arthritis with damp-heat obstruction syndrome. Guiding J Traditional Chin Med Pharm. (2013) 19:61–3. doi: 10.13862/j.cnki.cn43-1446/r.2013.02.020

[157] Hu RY Liu XS Liu JG Li JW Xiao ZG. Meta analysis of Danggui Niantong Tang in the treatment of rheumatoid arthritis with damp-heat obstruction syndrome. Gansu Med J. (2021) 40:133–8. doi: 10.15975/j.cnki.gsyy.2021.02.012

[158] Liang LY Lin HX Cai YS Lin SC Tan JW Ma GL. Effect of danggui niantongtang on intestinal flora in adjuvant-induced arthritis rats of wind-dampness-heat arthralgia:Based on 16S rDNA sequencing. Chin J Exp Traditional Med Formulae. (2023) 29:18–27. doi: 10.13422/j.cnki.syfjx.20230337

[159] Pal D Naskar M Bera A Mukhopadhyay B. Chemical synthesis of the pentasaccharide repeating unit of the O-specific polysaccharide from Ruminococcus gnavus. Carbohydr Res. (2021) 507:108384. doi: 10.1016/j.carres.2021.108384, PMID: 34229203

[160] Wang Y Yao W Li B Qian S Wei B Gong S. Nuciferine modulates the gut microbiota and prevents obesity in high-fat diet-fed rats. Exp Mol Med. (2020) 52:1959–75. doi: 10.1038/s12276-020-00534-2, PMID: 33262480 PMC8080667

[161] Xu H Huang Y Wang Y Tang F Yao XM Ma WK. Clinical observation on jinwu jiangu capsules combined with leflunomide in treatment for 38 cases of rheumatoid arthritis of cold-dampness obstruction type. J Traditional Chin Med. (2020) 61:607–12. doi: 10.13288/j.11-2166/r.2020.07.013

[162] Ling Y Xiao M Huang ZW Xu H Huang FQ Ren NN. Jinwujiangu capsule treats fibroblast-like synoviocytes of rheumatoid arthritis by inhibiting pyroptosis via the NLRP3/CAPSES/GSDMD pathway. Evidence-Based complementary Altern Med. (2021) 2021:4836992. doi: 10.1155/2021/4836992, PMID: 34853599 PMC8629621

[163] Lu D Huang Y Ma W Chen C Hou L. Jin-wu-jian-gu formulation attenuates rheumatoid arthritis by inhibiting the IL33-ST2 signaling pathway. Evidence-Based complementary Altern Med. (2022) 2022:6821388. doi: 10.1155/2022/6821388, PMID: 35096114 PMC8794654

[164] Mangalea MR Paez-Espino D Kieft K Chatterjee A Chriswell ME Seifert JA. Individuals at risk for rheumatoid arthritis harbor differential intestinal bacteriophage communities with distinct metabolic potential. Cell Host Microbe. (2021) 29:726–739.e5. doi: 10.1016/j.chom.2021.03.020, PMID: 33957082 PMC8186507

[165] Shi W Tang N Xiao J Zhang LJ Chai LM Wu JY. Clinical research on the treatment of active rheumatoid arthritis with lijie capsule. Lishizhen Med Materia Med Res. (2010) 21:3230–1.

[166] Sun HH. Study on the effect of Lijie capsule in treating the mice with collagen-induced arthritis from intestinal flora. China National Knowledge Infrastructure (CNKI): Guangxi University of Traditional Chinese Medicine (2020). doi: 10.27879/d.cnki.ggxzy.2020.000300

[167] Li X Xie Y Kang A Wang Y. New bitongling (NBTL) ameliorates rheumatoid arthritis in rats through inhibiting JAK2/STAT3 signaling pathway. Eur J Histochem. (2021) 65(1):3202. doi: 10.4081/ejh.2021.3202, PMID: 33634679 PMC7907992

[168] Guan Y Zhao X Lu Y Zhang Y Lu Y Wang Y. New bitongling regulates gut microbiota to predict angiogenesis in rheumatoid arthritis via the gut-joint axis: a deep neural network approach. . Front Microbiol. (2025) 16:1528865. doi: 10.3389/fmicb.2025.1528865, PMID: 39963498 PMC11830818

[169] Wang L Zhou CY Yang HM. Fang Dingya’s experience in the application of Erxian decoction in treating rheumatic diseases. Henan Traditional Chin Med. (2014) 34:1470–1. doi: 10.16367/j.issn.1003-5028.2014.08.020

[170] Yang CW Hou X. Q Zhang HJ. Clinical observation of Erxian Decoction on senile rheumatoid arthritis patients with bone osteoporosis. J Basic Chin Med. (2018) 24:1447–50. doi: 10.19945/j.cnki.issn.1006-3250.2018.10.033

[171] Liu J Li B Zhou X Liu G Li C Hu Z. Uncovering the mechanisms of Zhubi decoction against rheumatoid arthritis through an integrated study of network pharmacology, metabolomics, and intestinal flora. J Ethnopharmacol. (2025) 336:118736. doi: 10.1016/j.jep.2024.118736, PMID: 39186991

[172] Chu H Li XL. Jingfang Baidu san treatment of 20 cases of rheumatoid arthritis. J Pract Traditional Chin Internal Med. (2009) 23:69–70.

[173] Wang X Pan L Niu D Zhou J Shen M Zeng Z. Jingfang Granules alleviates the lipid peroxidation induced ferroptosis in rheumatoid arthritis rats by regulating gut microbiota and metabolism of short chain fatty acids. J Ethnopharmacol. (2025) 339:119160. doi: 10.1016/j.jep.2024.119160, PMID: 39608616

[174] Zhao J Zha Q Jiang M Cao H Lu A. Expert consensus on the treatment of rheumatoid arthritis with Chinese patent medicines. J Altern complementary Med (New York NY). (2013) 19:111–8. doi: 10.1089/acm.2011.0370, PMID: 22866945

[175] Li HF Zhong Q Ma WK Huang Y Zhu D Chen CM. Effect of Jinwu Jiangu Capsules on intestinal flora of collagen-induced arthritis model rats was analyzed based on 16S rRNA technology. Drugs Clinic. (2022) 37:2413–22.

[176] Liu Y. The effect of warm needle moxibustion combined with xinbitongling granules on the expression of TNF-α and IFN-γ in patients with rheumatoid arthritis. Asia-pacific Traditional Med. (2016) 12:141–2.

[177] Liu Y Wei G Lu TL Guo HY Wang Y. Effects of New Bitongling Compound on the T cell semi-group of CIA rats. China Med Herald. (2016) 13:8–11.

[178] Deng Y Chen Y Chen ZY Liu ZQ. Study on effect of tripterygium wilfordii on rheumatoid arthritis and igA, igG and RF. Chin Arch Traditional Chin Med. (2020) 38:234–6. doi: 10.13193/j.issn.1673-7717.2020.02.058

[179] Xu LX He DY. Research progress on the efficacy and mechanism of action of Tripterygium wilfordii and its derivatives in rheumatoid arthritis. Lishizhen Med Materia Med Res. (2022) 33:1703–6.

[180] Zhang Y Mao X Li W Chen W Wang X Ma Z. Tripterygium wilfordii: An inspiring resource for rheumatoid arthritis treatment. Medicinal Res Rev. (2021) 41:1337–74. doi: 10.1002/med.21762, PMID: 33296090

[181] Pan HD Xiao Y Wang WY Ren RT Liang LX Liu L. Traditional chinese medicine as a treatment for rheumatoid arthritis: from empirical practice to evidence-based therapy. Engineering. (2019) 5:178–202. doi: 10.1016/j.eng.2019.01.018

[182] Liu W Zhang YY. Effects of tripterygium glycosides on fibroblast-like synoviocyte α7 nicotinic acetylcholine receptor and inflammatory factors in patients with rheumatoid arthritis. Shandong J Traditional Chin. (2019) 38:1166–1170 + 1197. doi: 10.16295/j.cnki.0257-358x.2019.12.017

[183] Gao LX. Observation on the effect of tripterygium wilfordii polyglucoside combined with methotrexate tab lets in the treatment of interstitial lung disease in rheumatoid arthritis. J Bethune Med Sci. (2020) 18:143–5. doi: 10.16485/j.issn.2095-7858.2020.02.017

[184] Qin ZY Tan GY Jiang SQ Zhao ZX Huang M Jin J. Effects of Tripterygium Glycosides Tab lets on gut Microbiota in Rats with Rheumatoid Arthritis. China Pharmacist. (2022) 25:789–94. doi: 10.19962/j.cnki.issn1008-049X.2022.05.006

[185] Wang J Wang F. Research progress of total glucosides of paeony in treatment of autoimmune diseases. Med Recapitulate. (2021) 27:4481–5.

[186] Peng J Lu X Xie K Xu Y He R Guo L. Dynamic alterations in the gut microbiota of collagen-induced arthritis rats following the prolonged administration of total glucosides of paeony. Front Cell Infect Microbiol. (2019) 9204:204. doi: 10.3389/fcimb.2019.00204, PMID: 31245305 PMC6581682

[187] Manheimer E Wieland S Kimbrough E Cheng K Berman BM. Evidence from the cochrane collaboration for traditional chinese medicine therapies. J Altern complementary Med (New York NY). (2009) 15:1001–14. doi: 10.1089/acm.2008.0414, PMID: 19757977 PMC2856612

[188] Liu W Zhang Y Zhu W Ma C Ruan J Long H. Sinomenine inhibits the progression of rheumatoid arthritis by regulating the secretion of inflammatory cytokines and monocyte/macrophage subsets. Front Immunol. (2018) 9:2228. doi: 10.3389/fimmu.2018.02228, PMID: 30319663 PMC6168735

[189] Zhou H Liu JX Luo JF Cheng CS Leung EL Li Y. Suppressing mPGES-1 expression by sinomenine ameliorates inflammation and arthritis. Biochem Pharmacol. (2017) 142:133–44. doi: 10.1016/j.bcp.2017.07.010, PMID: 28711625

[190] Zhou H Wong YF Wang J Cai X Liu L. Sinomenine ameliorates arthritis via MMPs, TIMPs, and cytokines in rats. Biochem Biophys Res Commun. (2008) 376:352–7. doi: 10.1016/j.bbrc.2008.08.153, PMID: 18782565

[191] Jiang ZM Zeng SL Huang TQ Lin Y Wang FF Gao XJ. Sinomenine ameliorates rheumatoid arthritis by modulating tryptophan metabolism and activating aryl hydrocarbon receptor via gut microbiota regulation. Sci Bull. (2023) 68:1540–55. doi: 10.1016/j.scib.2023.06.027, PMID: 37422372

[192] Dong C Yu J Yang Y Zhang F Su W Fan Q. Berberine, a potential prebiotic to indirectly promote Akkermansia growth through stimulating gut mucin secretion. Biomed Pharmacother. (2021) 139:111595. doi: 10.1016/j.biopha.2021.111595, PMID: 33862492

[193] Han S Li LZ Song SJ. Daphne giraldii Nitsche (Thymelaeaceae): Phytochemistry, pharmacology and medicinal uses. Phytochemistry. (2020) 171:112231. doi: 10.1016/j.phytochem.2019.112231, PMID: 31901473

[194] Cheng ZL Li XH Yang Y. Analysis of the therapeutic effect of zushima tabl ets in the treatment of rheumatoid arthritis. Clin J Chin Med. (2011) 3:64–5.

[195] Tang Z Sun Q Ji H. Observation of the clinical effect of the combined therapy of zushima tab let and western medicine in the treatment of rheumatoid arthritis and MRI test results. Pak J Pharm Sci. (2019) 32:1415–8., PMID: 31551223

[196] Shan J Peng L Qian W Xie T Kang A Gao B. Integrated serum and fecal metabolomics study of collagen-induced arthritis rats and the therapeutic effects of the zushima tab let. Front Pharmacol. (2018) 9891:891. doi: 10.3389/fphar.2018.00891, PMID: 30154719 PMC6102586

[197] Hou M Wang R Zhao S Wang Z. Ginsenosides in Panax genus and their biosynthesis. Acta Pharm Sin B. (2021) 11:1813–34. doi: 10.1016/j.apsb.2020.12.017, PMID: 34386322 PMC8343117

[198] Guo LX Wang HY Liu XD Zheng JY Tang Q Wang XN. Saponins from Clematis mandshurica Rupr. regulates gut microbiota and its metabolites during alleviation of collagen-induced arthritis in rats. . Pharmacol Res. (2019) 149:104459. doi: 10.1016/j.phrs.2019.104459, PMID: 31541689

[199] Lin TF Wang L Zhang Y Zhang JH Zhou DY Fang F. Uses, chemical compositions, pharmacological activities and toxicology of Clematidis Radix et Rhizome- a Review. J Ethnopharmacol. (2021) 270:113831. doi: 10.1016/j.jep.2021.113831, PMID: 33476714

[200] Qin H Fu Y Zhou K Song H Fang G Chen Q. Toddalia asiatica extract attenuates adjuvant-induced arthritis by modulating colon Th17/Treg balance and colony homeostasis. J Ethnopharmacology. (2023) 313:116542. doi: 10.1016/j.jep.2023.116542, PMID: 37127142

[201] Yang D Lv G Wu Y Guo W Wang Y Hu J. Licorice-regulated gut-joint axis for alleviating collagen-induced rheumatoid arthritis. Phytomedicine. (2024) 135:156203. doi: 10.1016/j.phymed.2024.156203, PMID: 39510013

[202] Amir I Bouvet P Legeay C Gophna U Weinberger A. Eisenbergiella tayi gen. nov., sp. nov., isolated from human blood. Int J systematic evolutionary Microbiol. (2014) 64:907–14. doi: 10.1099/ijs.0.057331-0, PMID: 24282142

[203] O’Keefe SJ. Diet, microorganisms and their metabolites, and colon cancer. Nat Rev Gastroenterol Hepatol. (2016) 13:691–706. doi: 10.1038/nrgastro.2016.165, PMID: 27848961 PMC6312102

[204] Ye M Feng Q Jiang Y Wang F Shi X Yang X. Structure characterization and anti-rheumatoid arthritis activity of a polysaccharide from notopterygium incisum. Mol Nutr Food Res. (2023) 67:e2200713. doi: 10.1002/mnfr.202200713, PMID: 37143438

[205] Wu SS Xu XX Shi YY Chen Y Li YQ Jiang SQ. System pharmacology analysis to decipher the effect and mechanism of active ingredients combination from herb couple on rheumatoid arthritis in rats. J Ethnopharmacol. (2022) 288:114969. doi: 10.1016/j.jep.2022.114969, PMID: 34999146

[206] Zhang L Song P Zhang X Metea C Schleisman M Karstens L. Alpha-glucosidase inhibitors alter gut microbiota and ameliorate collagen-induced arthritis. Front Pharmacol. (2019) 101684:1684. doi: 10.3389/fphar.2019.01684, PMID: 32116681 PMC7010955

[207] Yang YN Wang QC Xu W Yu J Zhang H Wu C. The berberine-enriched gut commensal Blautia producta ameliorates high-fat diet (HFD)-induced hyperlipidemia and stimulates liver LDLR expression. BioMed Pharmacother. (2022) 155:113749. doi: 10.1016/j.biopha.2022.113749, PMID: 36174380

